# Endoplasmic reticulum stress regulators exhibit different prognostic, therapeutic and immune landscapes in pancreatic adenocarcinoma

**DOI:** 10.1111/jcmm.18092

**Published:** 2024-02-01

**Authors:** Shanshan Liu, Kaini He, Longbao Yang, Fangshi Xu, Xiaoguang Cui, Li Qu, Xueyi Li, Bin‐cheng Ren

**Affiliations:** ^1^ Department of Rheumatology and Immunology The Second Affiliated Hospital of Xi'an Jiaotong University Xi'an Shaanxi Province China; ^2^ Department of Gastroenterology The Second Affiliated Hospital of Xi'an Jiaotong University Xi'an Shaanxi Province China; ^3^ Department of Medicine Xi'an Jiaotong University Xi'an Shaanxi Province China

**Keywords:** endoplasmic reticulum stress, immune checkpoint blockade, immune microenvironment, pancreatic adenocarcinoma, prognosis, unfolded protein response

## Abstract

Endoplasmic reticulum stress (ERS) and unfolded protein response are the critical processes of tumour biology. However, the roles of ERS regulatory genes in pancreatic adenocarcinoma (PAAD) remain elusive. A novel ERS‐related risk signature was constructed using the Lasso regression analysis. Its prognostic value, immune effect, metabolic influence, mutational feature and therapeutic correlation were comprehensively analysed through multiple bioinformatic approaches. The biofunctions of KDELR3 and YWHAZ in pancreatic cancer (PC) cells were also investigated through colony formation, Transwell assays, flow cytometric detection and a xenograft model. The upstream miRNA regulatory mechanism of KDELR3 was predicted and validated. ERS risk score was identified as an independent prognostic factor and could improve traditional prognostic model. Meanwhile, it was closely associated with metabolic reprogramming and tumour immune. High ERS risk enhanced glycolysis process and nucleotide metabolism, but was unfavourable for anti‐tumour immune response. Moreover, ERS risk score could act as a potential biomarker for predicting the efficacy of ICBs. Overexpression of KDELR3 and YWHAZ stimulated the proliferation, migration and invasion of SW1990 and BxPC‐3 cells. Silencing KDELR3 suppressed tumour growth in a xenograft model. miR‐137 could weaken the malignant potentials of PC cells through inhibiting KDELR3 (5′‐AGCAAUAA‐3′). ERS risk score greatly contributed to PAAD clinical assessment. KDELR3 and YWHAZ possessed cancer‐promoting capacities, showing promise as a novel treatment target.

## 1 INTRODUCTION

Pancreatic adenocarcinoma (PAAD), a common abdominal cancer, is associated with high malignancy and mortality. According to the American Cancer Society, PAAD accounts for >400,000 deaths in the United States each year, contributing to 4.7% of all cancer‐related deaths.^1^ The 5‐year overall survival rate (OSR) of patients with PAAD is <35%.^2^ Approximately 10% of patients with PAAD are diagnosed with localized carcinoma and are eligible for surgical excision.^3^ The incidence of pancreatic cancer (PC) has been increasing.^3^ Recent advances in PAAD treatment have not improved the therapeutic outcomes. FOLFIRINOX (fluorouracil, leucovorin, irinotecan and oxaliplatin) and gemcitabine, which are the mainstream chemotherapy regimens for PAAD, have not increased the median disease‐free survival to >25 months.^4^ Additionally, programmed cell death protein 1/ligand 1 (PD‐1/L1) inhibitors such as pembrolizumab exert beneficial effects in <14.2% of patients.^5^ Thus, there is an urgent need to develop new therapeutic strategies and modify the currently used prognostic assessment system. Endoplasmic reticulum (ER) stress (ERS), a hallmark of cancer, is involved in tumour onset and progression.

Extrinsic and intrinsic factors, such as hypoxia, increased mutation rate, elevated demand for protein synthesis, and oncogene activation disrupt the homeostasis of protein synthesis in ER, which is called ER stress (ERS).^6^ To maintain proteostasis, cells activate a unique mechanism, termed the unfolded protein response (UPR). UPR can promote protein folding and enhance the clearance of misfolded proteins that have accumulated due to ERS.^7^ Additionally, UPR is regulated by three stress transducers, namely ATF6, EIF2AK3 and ERN1.^8^ ERS and UPR are reported to regulate tumour cell proliferation, progression, immune regulation, immunotherapy response and drug resistance.^9^ For example, the ERS‐related gene *SERP1* is associated with the prognosis and immune response in skin cutaneous melanoma.^10^ ERS‐induced exosomal miR‐27a‐3p promotes immune escape in breast cancer by regulating *CD274* expression in macrophages.^11^ These findings indicate that targeting ERS can be a potential therapeutic strategy for cancer.

KDELR3 belongs to the KDEL (Lys‐Asp‐Glu‐Leu) receptor (KDELR) family, whose members regulate protein transport between Golgi and ER.^12^ Mechanistically, KDELR members can bind to the heterotrimeric G protein in the Golgi complex, promoting initial protein transport.^13^ Previous studies have reported that KDELR3 functions as an adaptive gene in response to unfolded or misfolded protein accumulation in the ER. Thus, KDELR3 is a critical regulator of ERS and UPR and is associated with the pathogenesis of various human diseases, such as cancers. For example, a microarray study demonstrated that KDELR3 is a biomarker of atherosclerosis (AS) onset.^14^ In uveal melanoma (UM), KDELR3 is associated with prognosis, immune infiltration and chemoresistance.^15^ The roles of KDELR3 in PAAD have not been elucidated.

Several studies have elucidated the roles of ERS regulatory genes in cancer clinical assessment using high‐throughput approaches. Zhang et al. constructed an ERS signature to evaluate immune features in glioma.^16^ The prognostic value of ERS signature in various cancers, such as glioma,^17^ hepatocellular carcinoma (HCC),^18^ lung adenocarcinoma (LUAD)^19^ and renal cell carcinoma,^20^ has been previously evaluated. However, the clinical significance of ERS‐related genes in PAAD has not been well evaluated. This study comprehensively examined the roles of ERS‐related genes in the prognosis, tumour immune microenvironment (TIM), and mutational and metabolomic features of PAAD. To screen the candidates who can benefit from immune checkpoint blockade (ICB) therapy, the correlation of ERS risk score with the efficacy of ICB therapy was examined. Furthermore, the pro‐oncogenic roles of two core ERS signature genes, KDELR3 and YWHAZ, were verified using in vitro and in vivo experiments. A novel regulatory axis miR‐137/KDELR3 was also be proved to play critical functions in PAAD progression. The findings of this study can contribute to improve the clinical assessment and treatment of PAAD.

## 2 MATERIALS AND METHODS

### 2.1 Data source

The gene datasets and the basic information of patients are shown in Table 1. The clinical and transcriptomic data for bioinformatics analysis were obtained from The Cancer Genome Atlas (TCGA) (https://portal.gdc.cancer.gov/), The International Cancer Genome Consortium (ICGC) (https://dcc.icgc.org/releases) and Gene Expression Omnibus (GEO) (https://www.ncbi.nlm.nih.gov/geo/) databases. As TCGA‐PAAD cohort did not comprise unmatched non‐cancerous samples (*n* = 4), the data of 167 non‐cancerous pancreatic tissue samples were downloaded from the Genotype‐Tissue Expression (GTEx) database (https://xenabrowser.net/datapages/) to screen differentially expressed genes (DEGs). All transcriptome data were standardized using log2 (FPKM+1) transformation. The clinical characteristics of TCGA, ICGC and GEO cohorts are presented in Tables S1–S3.

**TABLE 1:** The purpose and description of the datasets used in this study.

Dataset	Available sample size (T/N)	Purpose
TCGA‐PAAD	178/4	To perform expression, prognostic, immune, metabolomic and ICB‐related analyses
GTEx	0/167	To provide the transcriptome data of non‐cancerous pancreatic samples
ICGC‐PACA‐AU	81/0	To validate the prognostic value of ERS risk signature
ICGC‐PACA‐CA	194/0	To serve as the prognostic validation cohort
GSE28735	84/45	To serve as the prognostic validation cohort
GSE57495	63/0	To serve as the prognostic validation cohort
GSE62452	69/61	To serve as the prognostic validation cohort
IMvigor 210	298/0	A real clinical cohort that provides the primary outcomes of patients with UC who received atezolizumab treatment was used to perform the ICB‐related analyses

Abbreviations: ERS, endoplasmic reticulum stress; GTEx, Genotype‐Tissue Expression; ICB, immune checkpoint blockade; ICGC, International Cancer Genome Consortium; PACA, pancreatic cancer; T/N, tumour samples versus non‐cancerous samples; TCGA, The Cancer Genome Atlas; UC, urothelial carcinomas.

### 2.2 Establishment of ERS‐related gene set

This study established a comprehensive ERS‐related gene set using the Molecular Signatures Database (MSigDB) (https://www.gsea‐msigdb.org/gsea/msigdb/). Some reviews^8^, ^9^, ^21^ have suggested that UPR is the major mechanism activated in response to ERS, which is mediated by ERN1, ATF6 and EIF2AK3. Hence, the following keywords were queried in the MSigDB: ‘Endoplasmic reticulum stress’, ‘Unfolded protein response’, ‘IRE1’, ‘ATF6’, and ‘PERK’. In total, 11 core gene sets were obtained (Table S4). After removing the overlapping genes, the final gene set comprised 379 ERS‐related genes (Table S5). To further confirm the reliability of the ERS gene set, biological functional analyses were performed using the Metascape online tool (http://metascape.org/).^22^

### 2.3 Construction of ERS‐related risk signature

The ERS‐related risk signature was constructed using a four‐step process. The ERS‐related DEGs were identified using the ‘Limma’ package in R software (Ver 4.1.2) based on the following criteria: adjusted *p <* 0.05 and |Log_2_ fold‐change (FC) ≥ 1|. Next, the ERS regulatory genes with prognostic value were identified using Cox univariate regression analysis. The DEGs were then intersected with prognostic genes using a Venn diagram. To ensure the accuracy of modelling, the genes whose expression patterns were inconsistent with their prognostic values were excluded. Finally, the genes obtained after these three screening rounds were subjected to least absolute shrinkage and selection operator (LASSO) regression analysis to construct a novel ERS risk signature for PAAD.

### 2.4 Gene Set Enrichment Analysis (GSEA)

GSEA was employed to unravel three issues. First, the differential UPRs between non‐cancerous and tumour samples were evaluated. Next, the relationships between ERS risk score and ERS intensity were explored. Third, the effects of ERS risk score on PAAD metabolic pathways, including glycolysis, amino acid (AA) metabolism, nucleotide metabolism and lipid metabolism, were examined. The detailed description of the candidate gene sets is provided in Table S6. GSEA was performed with 1000 permutations and the gene symbols parameter set to ‘No_collapse’. The phenotype labels were set as high‐risk score samples versus low‐risk score samples.

### 2.5 Survival analyses

Survival analyses were performed using the Kaplan–Meier method. The data of samples with a short follow‐up duration (<30 days) were excluded. The cohort was divided into high‐risk and low‐risk groups based on the optimal cut‐off value of the ERS risk score, which was determined using the Cutoff Finder online tool (http://molpath.charite.de/cutoff).^23^ Cox univariate and multivariate analyses were performed to identify the independent prognostic factors. The predictive accuracy of the ERS risk signature was evaluated using the receiver operating characteristic curve. Decision curve analysis (DCA) was performed to assess whether the ERS score can improve the traditional prognostic model based on TNM staging or clinical stage system. The corresponding modelling process relied on the multivariate logistic regression method. The clinical subgroup analyses were performed to map the applicable range of the ERS prognostic model. The data of patients with M1‐stage tumours were not included in the subgroup analyses owing to the limited number of samples (*n* = 5). A nomogram comprising TNM staging and ERS risk score was constructed to predict the 1‐year, 2‐year and 3‐year OSRs. The prognostic accuracy of the ERS signature was assessed using the calibration curves. The ICGC‐PACA‐CA, ICGC‐PACA‐AU, GSE62452, GSE28735 and GSE57495 datasets served as the validation cohorts.

### 2.6 Immune analyses

The infiltration levels of 22 immune cells in each PAAD sample were calculated using the cell‐type identification by estimating relative subsets of RNA transcripts (CIBERSORT) algorithm.^24^ The differential abundances of immune cells between the high‐risk and low‐risk groups were compared using the ‘Limma’ R package. The activities of 13 immune‐related pathways were quantified using single‐sample GSEA. ESTIMATE algorithm was used to measure the tumour purity and the stromal and immune cell admixture in each PAAD sample.^25^, ^26^ TIMER, a web server, was used for comprehensively analysing the tumour‐infiltrating immune cells (https://cistrome.shinyapps.io/timer/).^27^ The correlation between somatic copy number alterations (SCNAs) and the abundance of immune cells was determined.

### 2.7 Mutational analyses

The somatic mutation data of 149 PAAD samples were obtained from TCGA database. ‘Data Type’ and ‘Workflow Type’ were set to ‘Masked somatic mutation’ and ‘VarScan’, respectively. The tumour mutational burden (TMB) value of each PAAD sample was calculated as follows: TMB = total mutation frequency/38.^28^ The somatic mutation data were visualized and curated using the ‘maftools’ R package.^29^ The cBioPortal database (http://cbioportal.org) provided the mutational frequency and pattern of seven hub ERS‐related genes across four PC projects (*n* = 532 samples).^30^

### 2.8 Therapeutic correlation analyses

The correlation between the efficacy of ICB therapy and ERS risk score was analysed. TMB is a useful indicator for the selection of therapeutics for ICB therapy.^31^ A high TMB is correlated with favourable responses to ICB therapy. Hence, the differential TMBs between the high‐risk and low‐risk groups were determined. The tumour immune dysfunction and exclusion (TIDE) score predicts the response of patients to anti‐PD‐1/L1 and anti‐CTLA4 treatments based on the estimation of T‐cell dysfunction and tumour immune evasion.^32^ The TIDE score of each risk group was calculated using an online tool (http://tide.dfci.harvard.edu/login/). Patients exhibiting upregulated expression levels of immune checkpoints (ICs) exhibit favourable responses to ICB therapy.^33^, ^34^ Hence, the correlation between the expression levels of six critical ICs and the ERS risk score was analysed. T‐cell functions and cytotoxic effects are closely associated with the efficacy of immunotherapy.^35^, ^36^ Thus, the differential T‐cell functions and cytotoxic effects between the high‐risk and low‐risk groups were examined. ImmuCellAI can be used to predict the response to ICB therapy based on the abundance of 24 immune cells in samples (http://bioinfo.life.hust.edu.cn/).^37^ IMvigor210, a real clinical cohort, was used to examine the differential ERS risk scores in patients exhibiting differential outcomes to treatment with the PD‐1 inhibitor atezolizumab.^38^

### 2.9 Human Protein Atlas (HPA) database analysis

The HPA database provides the proteome profiles of 32 different cancers in the form of immunohistochemical images (https://www.proteinatlas.org/).^39^ The histological expression levels of seven hub ERS regulators in non‐cancerous and PC tissues were analysed.

### 2.10 Cell culture and transfection

In vitro experiments were performed with three PC cell lines (SW1990, BxPC‐3 and PANC‐1) and one healthy pancreatic cell line (HPDE6‐C7). 293T cells were used for mechanistic research of KDELR3. All cells were purchased from Procell Life Science & Technology Company (Wuhan, China). SW1990 cells were cultured in Leibovitz's L‐15 medium supplemented with 10% fetal bovine serum (FBS) and 1% penicillin/streptomycin. BxPC‐3 cells were cultured in Roswell Park Memorial Institute‐1640 medium, while PANC‐1, HPDE6‐C7 and 293 T cells were cultured in Dulbecco's modified Eagle's medium. The specific short hairpin RNA (shRNA) targeting two ERS signature genes (sh‐KDELR3 and sh‐YWHAZ) and their overexpression (OE‐KDELR3 and OE‐YWHAZ) lentivirus constructs were purchased from HanHeng Biotechnology (Shanghai, China). The specific sequences of these constructs are shown in Table S7. The cells were transfected with lentiviruses (HanHeng Biotechnology, Shanghai, China).

### 2.11 Quantitative real‐time polymerase chain reaction (qRT‐PCR)

Total RNA was extracted using chloroform and TRIzol reagent (TaKaRa, Japan). The optical density (OD) at 260 and 280 nm of the sample was measured using an ultraviolet spectrophotometer (Nanodrop 2000 spectrophotometer). An OD_260_ to OD_280_ ratio in the range of 1.8–2.0 indicates a high RNA purity. These RNA samples were subjected to further reverse transcription using the PrimeScript RT reagent kit (TaKaRa, Japan). qRT‐PCR analysis was performed using SYBR‐Green PCR Reagent (Takara, Japan) with the ABI Prism 7900 system. The PCR conditions were as follows: 95°C for 30 s (pre‐denaturation), followed by 40 cycles at 95°C for 5 s, 95°C for 15 s, 60°C for 30 s and 95°C for 15 s. *GAPDH* was used as the internal reference. The relative gene expression was calculated using the $2^{-\Delta \Delta C_{T}}$ method. The primer sequences used in qRT‐PCR analysis are shown in Table S8.

### 2.12 Immunohistochemical staining (IHC)

IHC was performed on four pairs of clinical samples from Second Affiliated Hospital of Xi'an Jiaotong University to validate expressions of KDELR3 and YWHAZ in PAAD. All patients signed informed consents before IHC detection. IHC process was analogous to a previous study.^40^ Briefly, paraffin sections was prepared through dehydration, embedding, dewaxing and rehydration. Subsequently, tissue sections were treated with 1.5% H_2_O_2_ solution to inactivate endogenous peroxidase. Citric acid buffer was used for antigen repair. After blocking by primary and secondary antibodies, tissue sections were stained by ABC method.

### 2.13 Flow cytometric analysis

Transfected cells were fixed with precooled 70% anhydrous ethanol at −20°C. Next, the cells were resuspended in phosphate‐buffered saline (PBS) and incubated with 100 μL RNase A (50 μL/mL) at 37°C for 30 min. The cells were then stained with propidium iodide (50 μg/mL, BD Biosciences) in the dark for 30 min. Flow cytometric analysis was performed using the Becton Dickinson FACScan system.

### 2.14 Colony formation assay

At 48 h post‐transfection, cells in the logarithmic growth phase were seeded into six‐well plates at a density of 1 × 10^3^/well. The colonies were fixed and stained with Giemsa stain in methanol. The number of colonies in five random visual fields was counted under the microscope.

### 2.15 Transwell migration and invasion assays

Transfected cells (1 × 10^4^ per well) were seeded into the 24‐well Transwell chambers (Corning, NY, USA). The medium supplemented with 0.1% FBS was added to the upper chambers, while the medium supplemented with 10% FBS was added to the lower chambers. After culturing for 24 h, the cells on the upper surface of the membrane were removed using PBS. The cells that invaded the membrane were fixed with paraformaldehyde and stained with 0.1% crystal violet. The number of cells in five random visual fields was counted under a high‐magnification microscope (100‐fold). The upper chambers were precoated with Matrigel to perform the invasion assays.

### 2.16 Dual‐luciferase assay

Dual‐luciferase assay was utilized to verify the miRNA binding sites with KDELR3. The recombinant vectors of wild type (WT) and mutation type (MUT) of KDELR3 3′UTR region were designed and synthesized by *Genechem* (Genechem Incorporation, Shanghai, China). The detailed protocol was similar to the previous description.^41^ Briefly, miRNA vectors and KDELR3 recombinant vectors were co‐transfected 293T cells. Then, luciferase reagent was added in lysed cells. The relative fluorescence intensity of each experimental group was measured using a microplate reader.

### 2.17 Xenograft assay

Female BALB/c nude mice aged 6 weeks were used to perform the tumour xenograft experiments. BxPC‐3 cells that were stably transfected with sh‐KDELR3 were subcutaneously injected into the flanks of each mouse. The tumour volume was calculated as follows: tumour volume = 0.5 × (tumour length) × (tumour width)^2^. Tumour length and width were measured using a vernier calliper once every 5 days. After 2 weeks, all mice were euthanized, and the xenograft tumours were excised. This study was approved by the Ethics Committee of the Second Affiliated Hospital of Xi'an Jiaotong University.

### 2.18 Statistical analysis

All statistical analyses were performed using the R software (version 4.1.2) and GraphPad Prism (version 8.0.1). The continuous variables between the groups were compared using the *t*‐test or Kruskal–Wallis test. Meanwhile, the categorical variables were compared using the Wilcoxon rank sum test. Correlation analyses were performed using the Spearman method. Prognostic meta‐analysis was performed using Review Manager 5.2 software (The Cochrane Collaboration, Oxford, UK) with the Mantel–Haenszel method. The odds ratio value was applied as the prognostic evaluation index. The *I* ^2^ value was used to assess the statistical heterogeneity of meta‐analysis. The fixed effect model was used if the *I* ^2^ value was <50%, while the random effect model if the *I* ^2^ value was ≥50%. The overall effects were tested using *Z*‐test. The in vitro experiments were repeated three times independently. Differences were considered significant at *p* < 0.05.

## 3 RESULTS

### 3.1 Establishment of a comprehensive and reliable ERS‐related gene set

**FIGURE 1:**
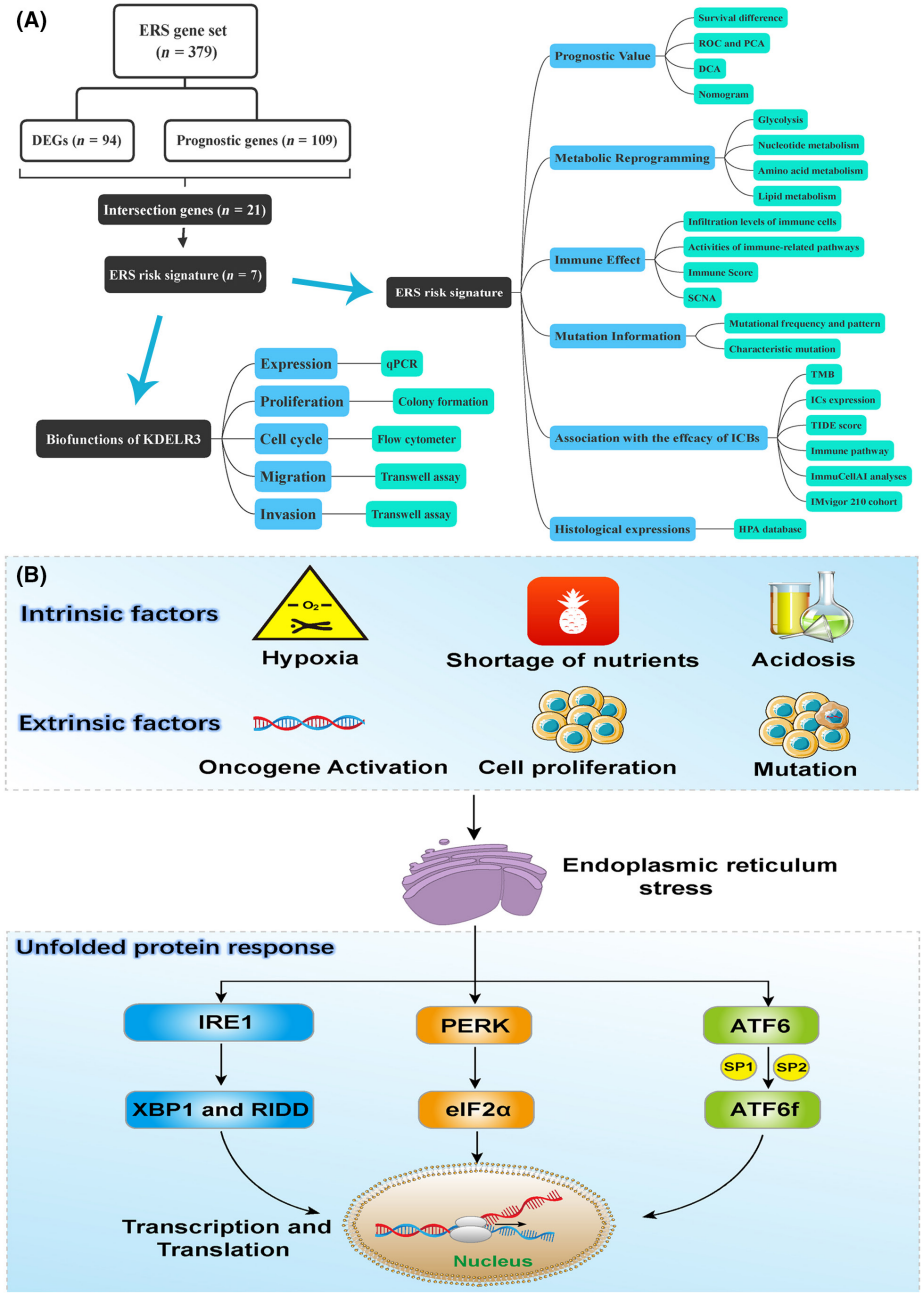
ERS is associated with cancer pathogenesis. (A) The flow chart of this study. (B) The core mechanism of ERS. ERS, endoplasmic reticulum stress.

The workflow of this study is shown in Figure 1A. An ERS‐related gene set was established based on some crucial reviews. The core mechanisms of UPR are shown in Figure 1B. Intrinsic (oncogene activation, high demand for cell proliferation, genomic instability and increased mutation frequency) and extrinsic (such as hypoxia, shortage of nutrients and acidosis) factors promote the accumulation of misfolded and unfolded proteins in ER. Subsequently, the folding of proteins and the clearance of unfolded proteins are upregulated through UPR, which is mediated by three major signalling pathways. The ERN1 pathway promotes the expression of transcription factors (TFs), especially XBP1.^42^ Additionally, ERN1 can directly target mRNAs and miRNAs through regulated IRE1‐dependent decay (RIDD), regulating the translation process.^43^ The EIF2AK3 pathway downregulates protein translation by phosphorylating EIF2A, which alleviates the burden of protein folding in ER.^21^ In the ATF6 pathway, ATF6 is transported to the Golgi apparatus and cleaved by the proteases SP1 and SP2. Subsequently, cleaved ATF6 releases its functional fragment ATF6f, which functions as a TF^44^ and promotes the degradation of misfolded proteins.

**FIGURE 2:**
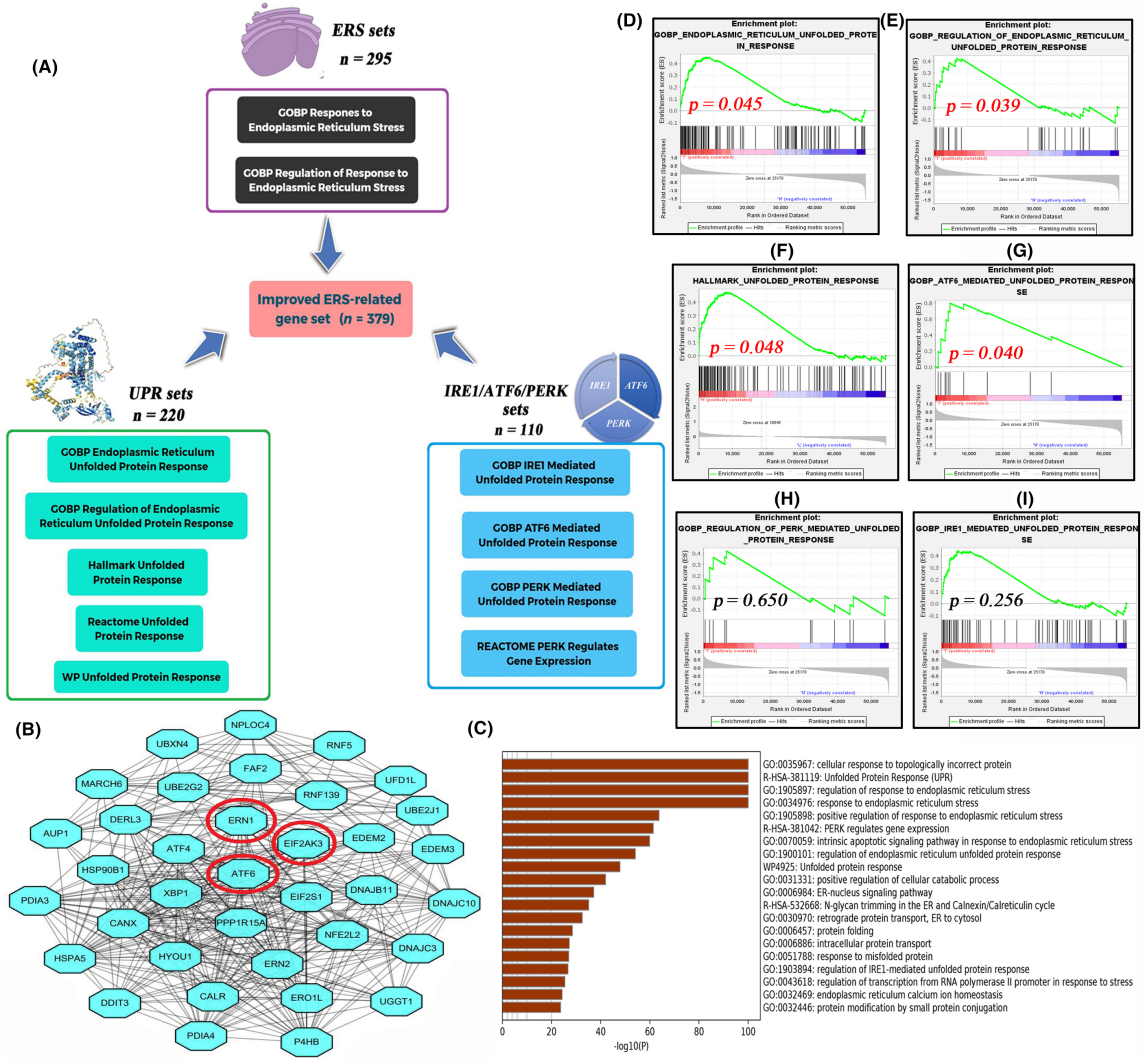
The construction of ERS‐related gene set. (A) A reliable and comprehensive ERS gene set was constructed based on 11 gene sets from the MSigDB. (B) The core module in the ERS network. (C) Biological function analyses of 379 ERS regulatory genes. (D–I) The enrichment results of ERS and three UPR pathways (TCGA‐PAAD cohort). ERS, endoplasmic reticulum stress; MSigDB, Molecular Signatures Database; UPR, unfolded protein response.

Based on the mechanisms of ERS and UPR, 11 gene sets were obtained from the MSigDB to establish a comprehensive ERS‐related gene set (Figure 2A). The protein–protein interaction (PPI) network of 379 ERS regulatory genes is shown in Figure S1A. Critical ERS‐related genes, such as *ERN1*, *EIF2AK3* and *ATF6*, were located in the core module (Figure 2B). Biological function analyses revealed that selected ERS‐related genes were enriched in ERS and UPR processes. These findings indicated that the ERS gene set established in this study was reliable (Figure 2C).

### 3.2 UPR is upregulated in PC samples

The differential activities of UPR between non‐cancerous and PC samples were analysed using GSEA. As shown in Figure 2D–I, the UPR pathways in PC samples were significantly upregulated when compared with those in non‐cancerous samples. In particular, only the ATF6 signalling pathway was markedly upregulated in PC samples (Figure 2G), whereas the ERN1 and EIF2AK3 pathways were unaffected (Figure 2H,I). Based on these findings, we hypothesized that the upregulation of UPR, especially the ATF6 pathway, is a biological hallmark of PAAD.

### 3.3 Construction of a novel ERS‐related risk signature for PAAD

**FIGURE 3:**
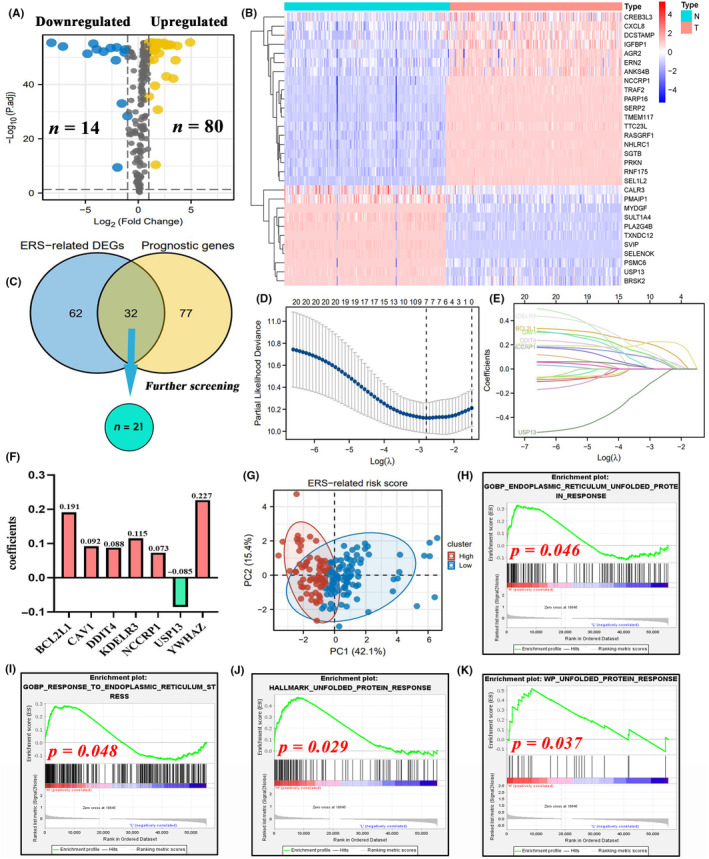
A novel ERS‐related risk signature. (A) Volcano plot shows 14 upregulated and 80 downregulated ERS‐related DEGs. (B) The heatmap of ERS‐related DEGs. (C) Venn diagram was used to obtain 21 intersecting ERS‐related genes. (D–E) The operation process of LASSO regression analysis. (F) The coefficients of 6 ERS‐related signature genes. (G) The PCA results of ERS risk signature. (H–K) The potential relationships between ERS risk score and factual ERS intensity. ERS, endoplasmic reticulum stress; DEGs, differentially expressed genes; LASSO, least absolute shrinkage and selection operator; PCA, principal component analysis.

Several ERS regulators (24.8%; 94/379) were differentially expressed between non‐cancerous and tumour samples (Figure 3A,B). Most of these DEGs were upregulated in PC samples. Approximately 30% of ERS‐related genes (28.8%; 109/379) were associated with the survival outcomes of patients with PAAD (Figure S1B). In total, 32 ERS‐related genes exhibited differential expression and had prognostic values (Figure 3C). The expression patterns of some intersection genes were not consistent with their prognostic values. For example, EEF2 was upregulated in tumour samples (Log2FC = 1.356) but predicted favourable prognosis (hazard ratio (HR) = 0.451). These ‘contradictory genes’ (*n* = 11) were not included in LASSO regression analysis to prevent their interference with modelling. As shown in Figure 3D, when partial‐likelihood deviance (PLD) was the minimum, the best value of Log(λ) was slightly larger than −3. At this time, the model fitting degree of ERS risk signature was optimal, and it was consisted of seven variables. Similarly, when Log(λ) took the optimum value (Around −3), there were only seven genes whose coefficients did not decay to 0 (Figure 3E).

Finally, a novel ERS‐related risk signature was constructed based on 22 core ERS‐related genes (Figure 3F). The risk score was calculated as follows (Figure 3H): ERS risk score = [(0.191 × *BCL2L1* relative expression) + (0.092 × *CAV1* relative expression) + (0.088 × *DDIT4* relative expression) + (0.115 × *KDELR3* relative expression) + (0.073 × *NCCRP1* relative expression) + (−0.085 × *USP13* relative expression) + (0.227 × *YWHAZ* relative expression). Principal component analysis (PCA) revealed that the ERS risk score can explain up to 57.5% of the prognostic variance, suggesting a good fit of the ERS model (Figure 3G).

Meanwhile, the associations of ERS risk score with the intensity of intracellular ER stress were investigated using GSEA method. The enrichments of ERS and UPR were significantly increased in PAAD samples with high ERS score, indicating that high ERS score was concomitant with high ERS intensity (Figure 3H–K).

### 3.4 Prognostic value of the ERS risk signature in PAAD

**FIGURE 4:**
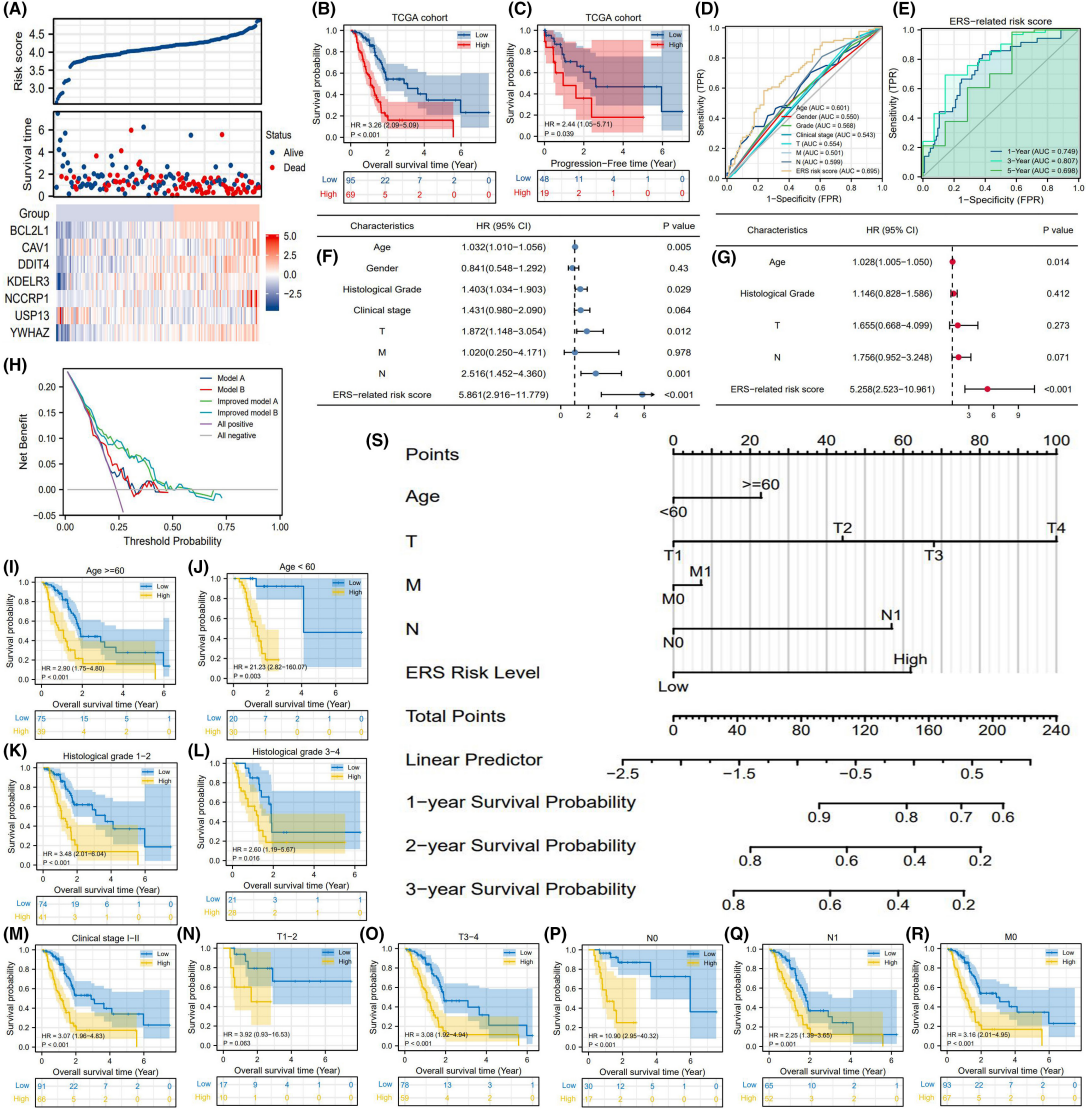
The prognostic value of ERS risk signature. (A) The risk plots of the ERS prognostic model. (B) The differential overall survival rates between the high‐risk and low‐risk groups. (C) The differential progression‐free survival rates between the high‐risk and low‐risk groups. (D) The accuracy of the ERS risk score and other clinical features for predicting OSR. (E) Time‐dependent accuracy of the ERS risk score for predicting OSR. (F, G) The identification of independent prognostic factors for PAAD using Cox univariate (blue) and multivariate (red) analyses. (H) The DCA results. Different curves represent four prognostic models based on multivariate logistic regression analysis. Model A comprises age, gender and clinical stage. Model B comprises age, gender and TNM staging. Improved model A represents model A with the ERS risk score. Improved model B represents model B with the ERS risk score. (I–R) The differential survival rates between clinical subgroups. (S) The nomogram comprising age, TNM staging and ERS risk score. DCA, decision curve analysis; ERS, endoplasmic reticulum stress; OSR, overall survival rate; PAAD, pancreatic adenocarcinoma.

Based on the optimal cut‐off value of the ERS risk score (4.185), 154 patients with PAAD in TCGA cohort were divided into high‐risk and low‐risk groups (Figure 4A). Patients in the high‐risk group were associated with poor overall survival outcomes (HR = 3.26, *p* < 0.01, Figure 4B) and decreased progression‐free survival (PFS) (HR = 2.44, *p* = 0.39, Figure 4C). In contrast to other clinicopathological characteristics (age, histological grade, gender, clinical stage and TNM staging), the ERS risk score exhibited an enhanced prediction accuracy (area under the curve (AUC) = 0.695, Figure 4D). Additionally, the ERS risk score exhibited the best performance for predicting 3‐year OSR (AUC = 0.807, Figure 4E). Univariate Cox analysis indicated that age, histological grade, TN stages and ERS risk score were associated with the prognosis of patients with PAAD (Figure 4F). ERS risk score (HR = 5.258) and age (HR = 1.028) were demonstrated to be the independent prognostic factors for PAAD (Figure 4G). Furthermore, the ERS risk score could significantly improve the clinical decision benefit of traditional prognostic models including AJCC‐Stage and TNM staging (Figure 4H). ROC analyses further indicated that ERS risk score could markedly improve the accuracy of TNM or AJCC‐Stage systems for predicting the survival outcomes of PC patients (Figure S1C–F). This suggested that the ERS risk score complemented the prognostic assessment of PAAD.

The ERS risk score could predict the prognosis of patients with different stages of PAAD. In particular, the ERS score could predict the differential survival rates between the high‐risk and low‐risk groups in most clinical subgroups, except in patients with T1‐2 stage tumours (Figure 4I–R). To enable clinical application, a nomogram comprising age, TNM staging and ERS risk score was constructed to predict the 1‐year, 2‐year and 3‐year survival rates of patients with PAAD (Figure 4S). For example, the 2‐year OSR of a 50‐year‐old patient who was diagnosed as T2N0M0 was predicted to be approximately 80% when the patient was classified into the low‐risk group. In contrast, the 2‐year OSR of this patient was predicted to be <60% when the patient was classified into the high‐risk group. The calibration plots indicated that the predicted survival rates were consistent with the actual survival rates (Figure S2).

### 3.5 ERS risk signature can be adapted to multiple external cohorts

**FIGURE 5:**
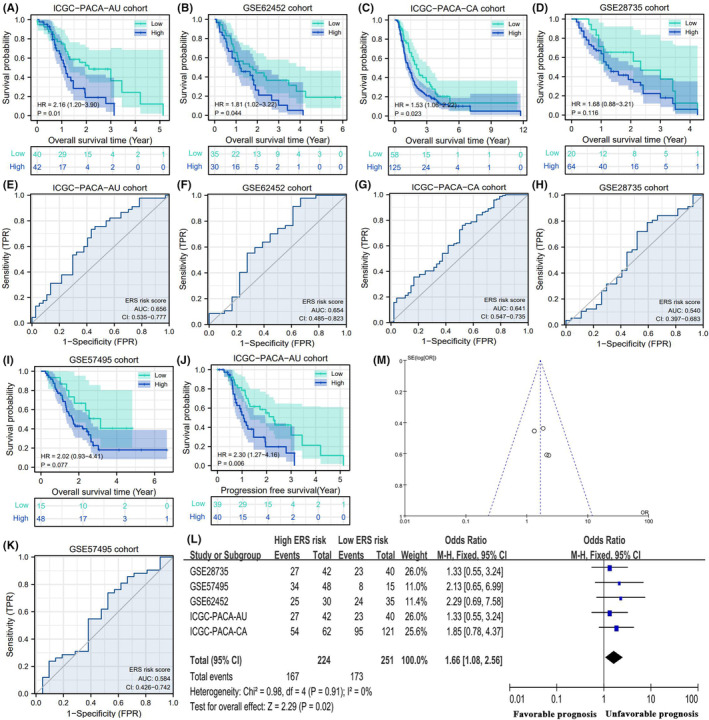
Confirmation of the prognostic value of ERS risk signature in multiple validation cohorts. (A–D, I) The differential overall survival rates between the ICGC‐PACA‐AU, GSE62452, ICGC‐PACA‐CA, GSE28735 and GSE57495 cohorts. (J) The PFS of ICGC‐PACA‐CA cohort. (E–H, K) The accuracy of ERS risk signature in predicting OSR in multiple validation cohorts. (L) Survival meta‐analysis of five validation cohorts. (M) The adjusted funnel plots. ERS, endoplasmic reticulum stress; ICGC, International Cancer Genome Consortium; OSR, overall survival rate; PFS, progression‐free survival.

To enable the application of the ERS risk signature, the prognostic value of the ERS risk signature was validated in five external cohorts. The ERS risk score could effectively stratify the prognosis of patients in the ICGC‐PACA‐AU (HR = 2.16, *p* = 0.01), GSE62452 (HR = 1.81, *p* = 0.044) and ICGC‐PACA‐CA (HR = 1.53, *p* = 0.023) cohorts. A high‐risk score indicated unfavourable survival outcomes (Figure 5A–C) and decreased PFS (Figure 5J). The predictive accuracy of the ERS model was moderate in these cohorts (AUC = 0.641–0.656) (Figure 5E–G). The prognostic values of the ERS model were not observed in the GSE28735 and GSE57495 cohorts (Figure 5D,H,I,K). Survival meta‐analysis was performed to comprehensively evaluate the prognostic value of the ERS risk score in five validation cohorts. As shown in Figure 5L, the overall mortality risk in the high‐risk group was markedly higher than that in the low‐risk group (*Z* = 2.29, *p* = 0.02). Although the ERS risk signature did not exhibit prognostic values in two validation cohorts, the overall effects of the ERS risk signature were closely associated with PAAD prognosis. The adjusted funnel plot suggested no publication bias (Figure 5M).

### 3.6 ERS risk score indicates metabolic changes, especially glycolysis and nucleotide metabolism

**FIGURE 6:**
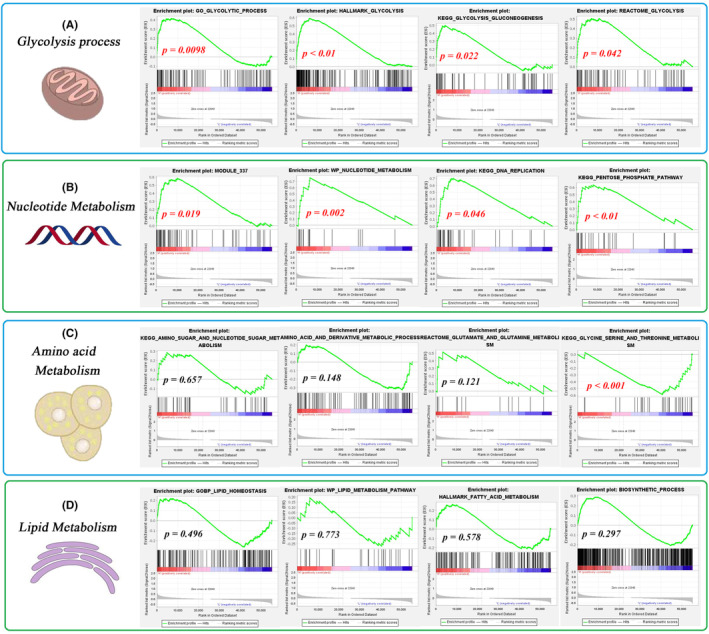
ERS risk score predicts metabolic reprogramming. (A) The differential enrichment of glycolysis in different risk groups. (B) The effects of ERS risk levels on nucleotide metabolism. (C) The effects of ERS risk levels on amino acid metabolism. (D) The enrichment of lipid metabolism was not markedly different between the groups. ERS, endoplasmic reticulum stress.

Metabolic changes are the core hallmarks of cancers. As shown in Table 2, aerobic glycolysis, nucleotide metabolism, AA metabolism and lipid metabolism are involved in tumour progression processes, such as cell proliferation and therapy resistance. This study investigated the effects of ERS risk levels on various metabolic pathways. The metabolic reprogramming toward glycolysis, also called the ‘Warburg effect’, indicates the preference of tumour cells for inefficient glucose utilization via glycolysis rather than oxidative phosphorylation.^45^ GSEA revealed that four glycolysis‐related genes were significantly enriched in the high‐risk group (Figure 6A). This study also analysed the correlation between the ERS risk score and four rate‐limiting enzymes of glycolysis. The expression levels of PKM, HK1 and HK2 in the high‐risk group were significantly higher than those in the low‐risk group (Figure S3A). However, the PFKFB3 expression levels were not significantly different between the high‐risk and low‐risk groups. The ERS risk score was positively correlated with the expression levels of PKM, HK1 and HK2 (Figure S3B–D) but not with those of PFKFB3 (Figure S3E). Thus, a high ERS risk score may be correlated with active glycolysis and the upregulation of relevant rate‐limiting enzymes.

**TABLE 2:** Correlation between ERS risk score and metabolic reprogramming.

Metabolic process	Enrichment score	Affected gene sets	Function in cancer
Glycolysis	0.415	GO glycolytic process	Active glycolysis can meet the metabolic requirements of tumour cell proliferation and confer therapeutic resistance
	0.591	Hallmark glycolysis	
	0.499	KEGG glycolysis Gluconeogenesis	
	0.507	Reactome glycolysis	
Nucleotide metabolism	0.584	MODULE 337	Nucleotides are the raw materials for nucleic acid synthesis and consequently support cell proliferation. Purine nucleotides promote the recognition of tumour cells with MHC class I expression by NK cells
	0.758	WP Nucleotide metabolism	
	0.703	KEGG DNA replication	
	0.637	KEGG pentose phosphate pathway	
Amino acid metabolism	−0.589	KEGG glycine, serine and threonine metabolism	Glycine and serine provide the essential precursors for the synthesis of proteins, nucleic acids and lipids that are required for tumour growth
Lipid metabolism	NA	NA	Lipids, especially cholesterol, promote membrane trafficking and signal transduction and mediate various cancer‐related signalling pathways through the lipid rafts

Abbreviations: ERS, endoplasmic reticulum stress; GO, Gene Ontology; KEGG, Kyoto Encyclopedia of Genes and Genomes; MHC, major histocompatibility complex; NA, not available; NK, natural killer; PAAD, Pancreatic adenocarcinoma.

Active nucleotide metabolism, which contributes to the occurrence and progression of tumours,^46^ was upregulated in the high‐risk group (Figure 6B). The enrichment of AA metabolism and lipid metabolism was not markedly different between the high‐risk and low‐risk groups (Figure 6C,D). Thus, nucleotide metabolism, but not AA metabolism and lipid metabolism, is upregulated in the high‐risk group.

### 3.7 Differential landscapes of TIM between the high‐risk and low‐risk groups

**FIGURE 7:**
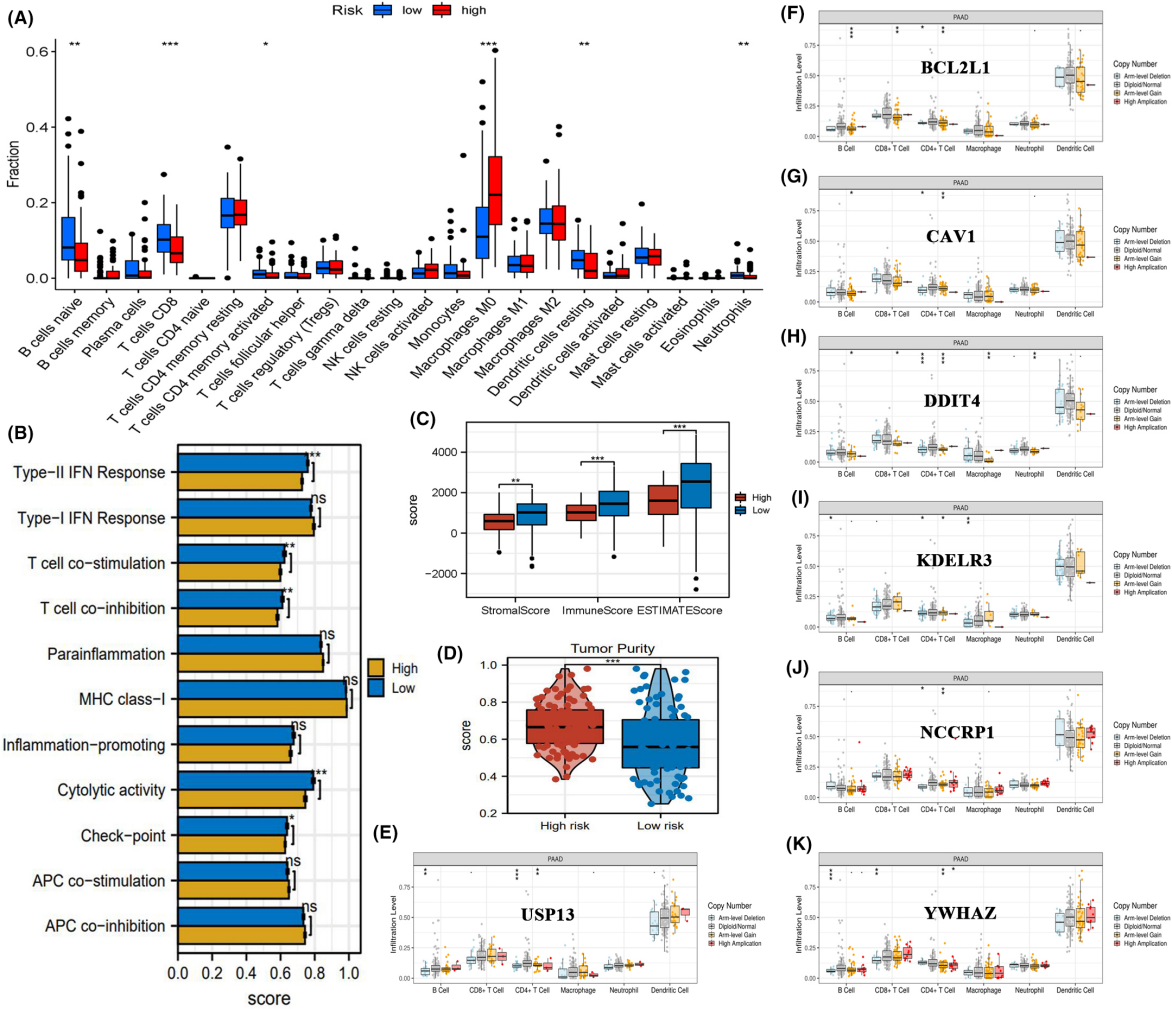
Tumour immune microenvironment varies between the ERS risk groups. (A) The differential infiltration levels of 22 lymphocyte subtypes between the high‐risk and low‐risk groups. (B) The differential activities of 11 immune‐related pathways between the high‐risk and low‐risk groups. (C) The differential immune scores between the high‐risk and low‐risk groups. (D) The differential tumour purity between the high‐risk and low‐risk groups. (E‐K) The correlations between the SCNA of ERS risk signature genes and the infiltration levels of six pivotal immune cells. APC, antigen‐presenting cells; ERS, endoplasmic reticulum stress; IFN, interferon; SCNA, somatic copy number alteration. *p < 0.05, **p < 0.01, ***p < 0.001.

The abundances of 22 immune cells in each PAAD sample are shown in Figure S4A. Immune cell enrichments significantly varied between the high‐risk and low‐risk groups. The levels of infiltrating B cells, CD8+ T cells, activated memory CD4+ T cells, activated dendritic cells and neutrophils were downregulated, whereas those of macrophages were upregulated in the high‐risk group (Figure 7A). As shown in Table 3, these changes in the abundance of immune cells suppressed anti‐tumour immune responses and promoted immune tolerance. For example, CD8+ T cells function as the most powerful effectors in the anti‐cancer immune response and are the targets for cancer immunotherapy.^47^ Hence, the downregulation of CD+ T cells inhibits the anti‐tumour immune process.

**TABLE 3:** The immune microenvironment profiles of the high‐risk group.

Immune cells	Changing trend	Basic function	Final effect on anti‐tumour immune response
B cells naïve	Decreased	Naive B cells refer to B cells that have not been activated by antigens. B cells present tumour antigens to T cells	Unfavourable
T cells CD8	Decreased	CD8+ T cells clear tumour cells through perforin‐granzyme and Fas–Fasl pathways	Unfavourable
T cells CD4 memory activated	Decreased	Memory CD4 T cells can rapidly enhance the anti‐tumour activity of CTLs	Unfavourable
Macrophages M0	Increased	Tumour macrophages can suppress T‐cell recruitment and function	Unfavourable
Dendritic cells resting	Decreased	DCs are the critical antigen‐presenting cells and recognize the tumour antigen	Unfavourable
Neutrophils	Decreased	Neutrophils promote tumour initiation by releasing ROS and RNS. Additionally, neutrophils can inhibit the functions of NK and CD8+ T cells	Beneficial

Abbreviations: CTLs, cytotoxic T lymphocytes; DCs, dendritic cells; ERS, endoplasmic reticulum stress; NK, natural killer; PAAD, pancreatic adenocarcinoma; RNS, reactive nitrogen species; ROS, reactive oxygen species.

Among the immune‐related signalling pathways, the activities of type‐II interferon (IFN) response, T‐cell functions, cytolytic activity and checkpoint were suppressed in the high‐risk group (Figure 7B). Type‐II IFN is a pleiotropic molecule with anti‐proliferative and pro‐apoptotic properties.^48^ The suppression of type‐II IFN leads to unfavourable anti‐tumour immune responses. Similar findings were observed in ‘ESTIMATE’ analyses. The stromal, immune and ESTIMATE scores in the high‐risk group were markedly lower than those in the low‐risk group (Figure 7C). The tumour purity in the high‐risk group was significantly higher than that in the low‐risk group, which can be attributed to the suppression of anti‐tumour immune responses in the high‐risk group (Figure 7D). The SCNAs of seven ERS signature genes were correlated with the infiltration levels of CD4+ T cells, CD8+ T cells and macrophages (Figure 7E–K). Thus, a high ERS risk score indicates the suppression of anti‐tumour responses. The immune heatmaps of different ERS risk groups are shown in Figure S4B.

### 3.8 *KRAS* , *TP53* , *SMAD4* and *CDKN2A* mutation frequencies are upregulated in the high‐risk group

**FIGURE 8:**
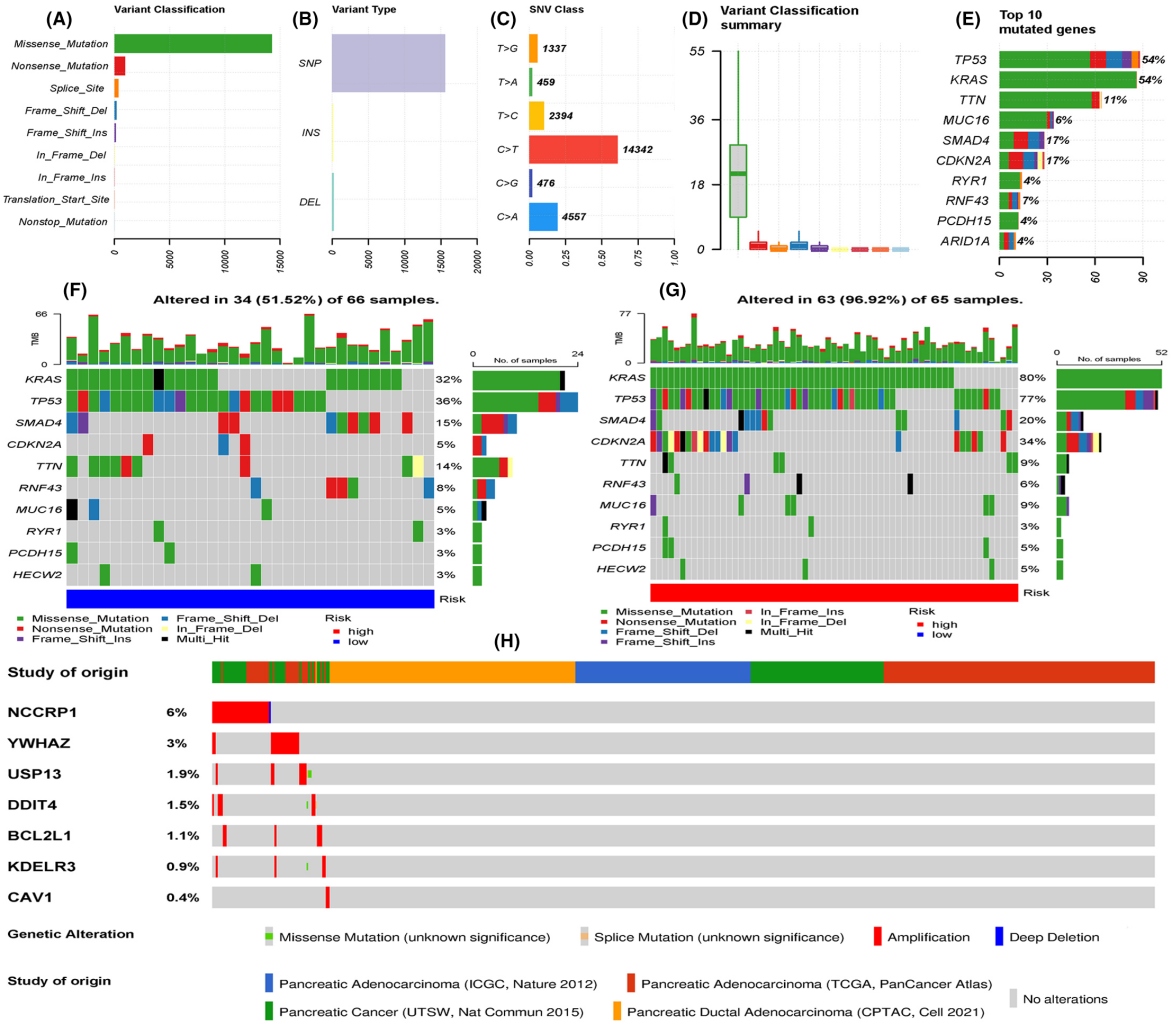
The mutation profile of the ERS risk signature genes. (A–D) The general mutation profiles of 131 PAAD samples in TCGA database, including mutational frequency and type. (E) The top 10 mutated genes in 131 PAAD samples. (F, G) The mutational waterfall plots of high‐risk and low‐risk groups. The top column shows the TMB in each PAAD sample. The characteristic mutated genes are listed on the left side of the plots, while their mutational frequencies are listed on the right side. (H) The somatic mutation frequency of ERS signature genes based on the cBioPortal database. ERS, endoplasmic reticulum stress; PAAD, pancreatic adenocarcinoma; TCGA, The Cancer Genome Atlas; TMB, tumour mutation burden.

This study investigated the transcriptomic information of ERS signature genes. The somatic mutation features of PC are shown in Figure 8A–D. The mean number of variants in each PC sample was as high as 25.5. Missense mutation was the most frequent alteration. Single nucleotide polymorphism (SNP) was the predominant variant, while insertion (INS) and deletion (DEL) were not common in PC samples. C>T (*n* = 14,323) and C>A (*n* = 4557) substitutions were the major SNP types. More than half of the PC samples exhibited *KRAS* and *TP53* mutations (Figure 8E). This suggests that *KRAS* and *TP53* are the characteristic mutation markers of PAAD.

The ERS risk score was closely related to the mutation frequencies of some oncogenes. In the high‐risk group, 96.62% of PAAD samples harboured at least one mutation in one gene. In contrast, only 51.52% of samples in the low‐risk group harboured at least one mutation in one gene. Furthermore, the mutation frequencies of *KRAS*, *TP53*, *SMAD4* and *CDKN2A* in the high‐risk group were higher than those in the low‐risk group (Figure 8F,G). As shown in Table 4, the upregulation of these genetic mutations promotes the malignant behaviours of PAAD, which may be the intrinsic reason for the poor prognosis of the high‐risk group. The mutation profile of ERS signature genes was conserved across multiple PC projects (Figure 8H). The mutation frequencies of these genes were less than 10%.

**TABLE 4:** The clinical implications of characteristic mutations in multiple cancers.

Mutation	Main study	Cancer type	Clinical implication
*KRAS*	PMID: 32725342	CRC, LUAD, PC	Worse prognosis
*TP53*	PMID: 29242642	OC, BC, GC	Therapy approach
*SMAD4*	PMID: 23139211	CRC	Biomarker of metastasis
*CDKN2A*	PMID: 27287845	Melanoma	Worse prognosis

Abbreviations: BC, breast cancer; CRC, colorectal cancer; GC, gastric cancer; LUAD, lung adenocarcinomas; OC, ovarian cancer; PC, pancreatic cancer.

### 3.9 ERS risk score is a potential biomarker for predicting ICB therapy response

**FIGURE 9:**
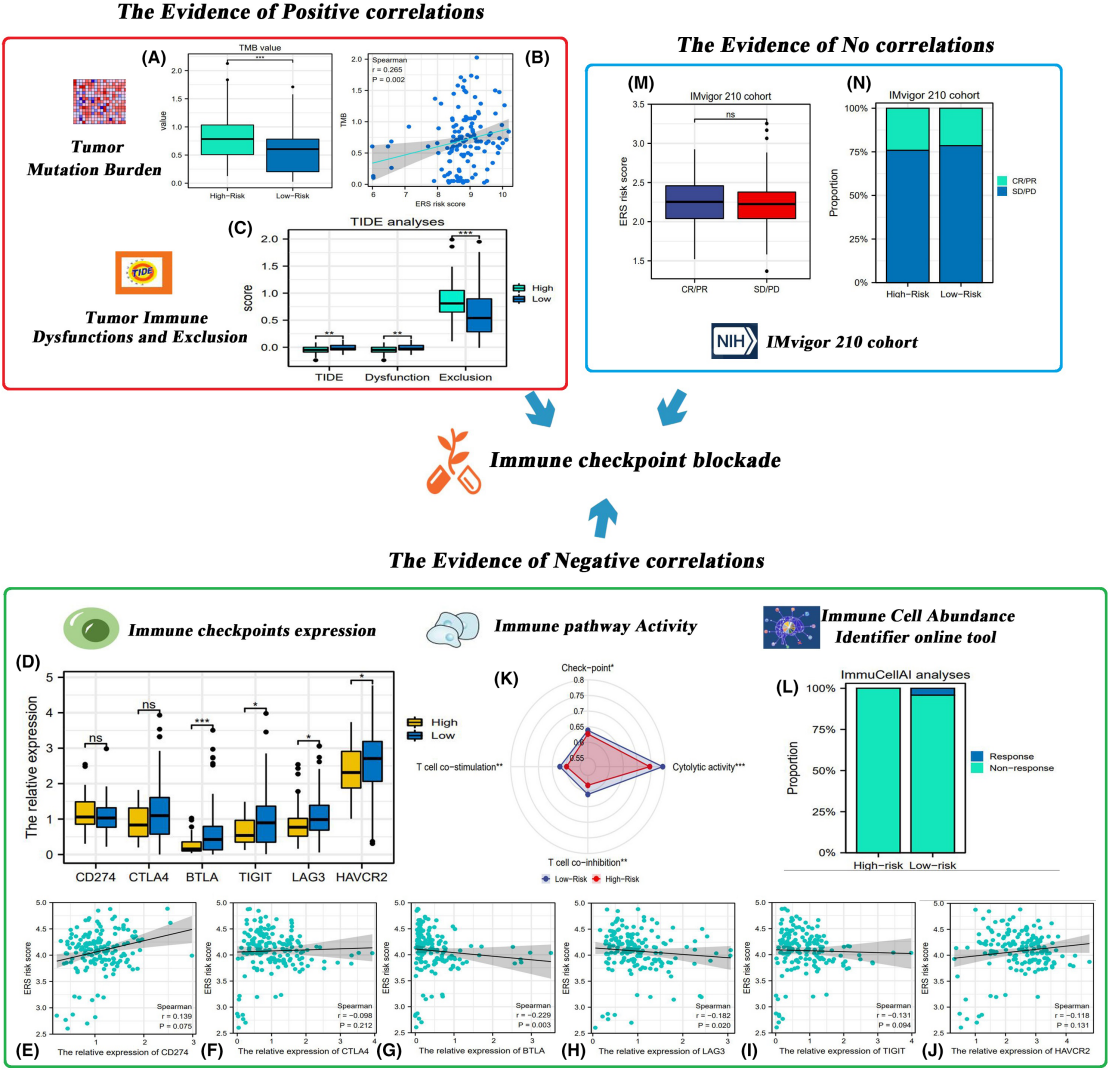
The potential correlation between ERS risk score and the efficacy of ICB therapy. (A) The differential TMB between the high‐risk and low‐risk groups. (B) The correlation between TMB and ERS risk score was analysed using the Spearman method. (C) The differential TIDE scores between the high‐risk and low‐risk groups. (D) The differential expression levels of ICs between the high‐risk and low‐risk groups. (E–J) The correlation of IC expression levels with ERS risk score. (K) The differential activities of four IC‐related immune pathways. (L) The ICB therapy response rate in different risk groups was predicted using the ImmuCellAI online tool. (M) The differential risk scores between the responder and non‐responder groups in the IMvigor 210 cohort. (N) The response rates in different risk groups. ERS, endoplasmic reticulum stress; ICs, immune checkpoints; ICB, immune checkpoint blockade; TMB, tumour mutation burden; TIDE, tumour immune dysfunction and exclusion. *p < 0.05, **p < 0.01, ***p < 0.001.

ICB therapy offers a novel paradigm for cancer treatment. Bioinformatic analyses revealed the potential correlation between ERS risk score and the efficacy of ICB therapy. The correlation patterns varied depending on the analysis (Figure 9). A high ERS risk score indicated a good response to ICB therapy. As shown in Figure 9A,B, the TMB in the high‐risk group was significantly higher than that in the low‐risk group and was positively correlated with the ERS risk score. A high TMB results in the production of tumour neoantigens and consequently activates the immune system to recognize tumour cells. Thus, patients with high TMB benefit from ICB therapy.^49^, ^50^ The TIDE score in the high‐risk group was markedly lower than that in the low‐risk group (Figure 9C). This suggests that patients in the high‐risk group are less likely to exhibit T‐cell dysfunction and immune evasion (Figure 9C).

A high ERS risk score was associated with unfavourable ICB therapy response (Figure 9D–J). The expression levels of BTLA, TIGIT, LAG3 and HACR2, but not CD274 and CTLA4, in the high‐risk group were downregulated when compared with those in the low‐risk group (Figure 9D). Additionally, the expression levels of BTLA, TIGIT, LAG3 and HACR2 were negatively correlated with the ERS risk score (Figure 9E–J). The upregulation of ICs is considered to be a marker of favourable ICB therapy response.^33^, ^51^ Thus, patients with a high‐risk score may not be sensitive to ICB therapy. Moreover, the functions of immunotherapy‐related signalling pathways, including ‘checkpoint’, ‘T‐cell functions’ and ‘cytolytic activity’, were downregulated in the high‐risk group, resulting in unfavourable ICB therapy response (Figure 9K). The predictive results of the online tool ‘ImmuCellAI’ were consistent with these findings. Patients in the high‐risk group were predicted to exhibit unfavourable ICB therapy responses. In contrast, the predicted response rate in the low‐risk group was 4.2% (4/95) (Figure 9L).

A real clinical cohort (IMvigor 210) was used to further investigate the correlation between ERS risk score and the efficacy of ICB (atezolizumab) therapy (Figure 9M,N). The ERS risk score was not significantly different between the responder and non‐responder groups (Figure 9M). The clinical response rates were similar between the high‐risk (24.2%) and low‐risk (21.5%) groups (Figure 9N).

### 3.10 ERS signature genes are differentially expressed in PC tissues

The histological expression levels of seven ERS signature genes were confirmed using the HPA database (Figure S5A). The BCL2L1, CAV1, KDELR3 and YWHAZ expression levels were upregulated in the tumour tissues and downregulated in the healthy pancreatic tissues. However, NCCRP1 was upregulated in tumour tissues and not detected in healthy tissues. This was not consistent with the mRNA expression patterns of these proteins. Furthermore, USP13 was downregulated in both cancer and healthy tissues.

### 3.11 KDELR3 promotes malignant behaviours and modulates the cell cycle of PC cells

Several studies have examined the roles of five ERS signature genes in PAAD (Table 5). However, limited studies have examined the roles of KDELR3, USP13 and YWHAZ. KDELR3 and YWHAZ were selected for further analysis as they exhibited the high coefficient values in the ERS risk model (0.115 and 0.227).

**TABLE 5:** Functions of ERS signature genes in PAAD.

Gene	Study (PMID)	Cancer type	Functions
*BCL2L1*	32424151	PAAD	Exhibits anti‐apoptotic activity and mediates the differentiation of pancreatic cancer cells
*CAV1*	34320244	PAAD	Upregulates glycolysis in pancreatic cancer cells and triggers cachectic states
*DDIT4*	34354858	PAAD	Mediates the anti‐proliferation effects of doxycycline and gemcitabine
*NCCRP1*	33414811	PAAD	Mediates ubiquitination in pancreatic cancer and is associated with poor survival outcomes of patients with PAAD
*USP13*	NA	PAAD	Unclear
*YWHAZ*	30519576	PAAD	Mediates tumour‐stroma crosstalk
*KDELR3*	NA	PAAD	Unclear

Abbreviations: ERS, endoplasmic reticulum stress; PAAD, pancreatic adenocarcinoma.

**FIGURE 10:**
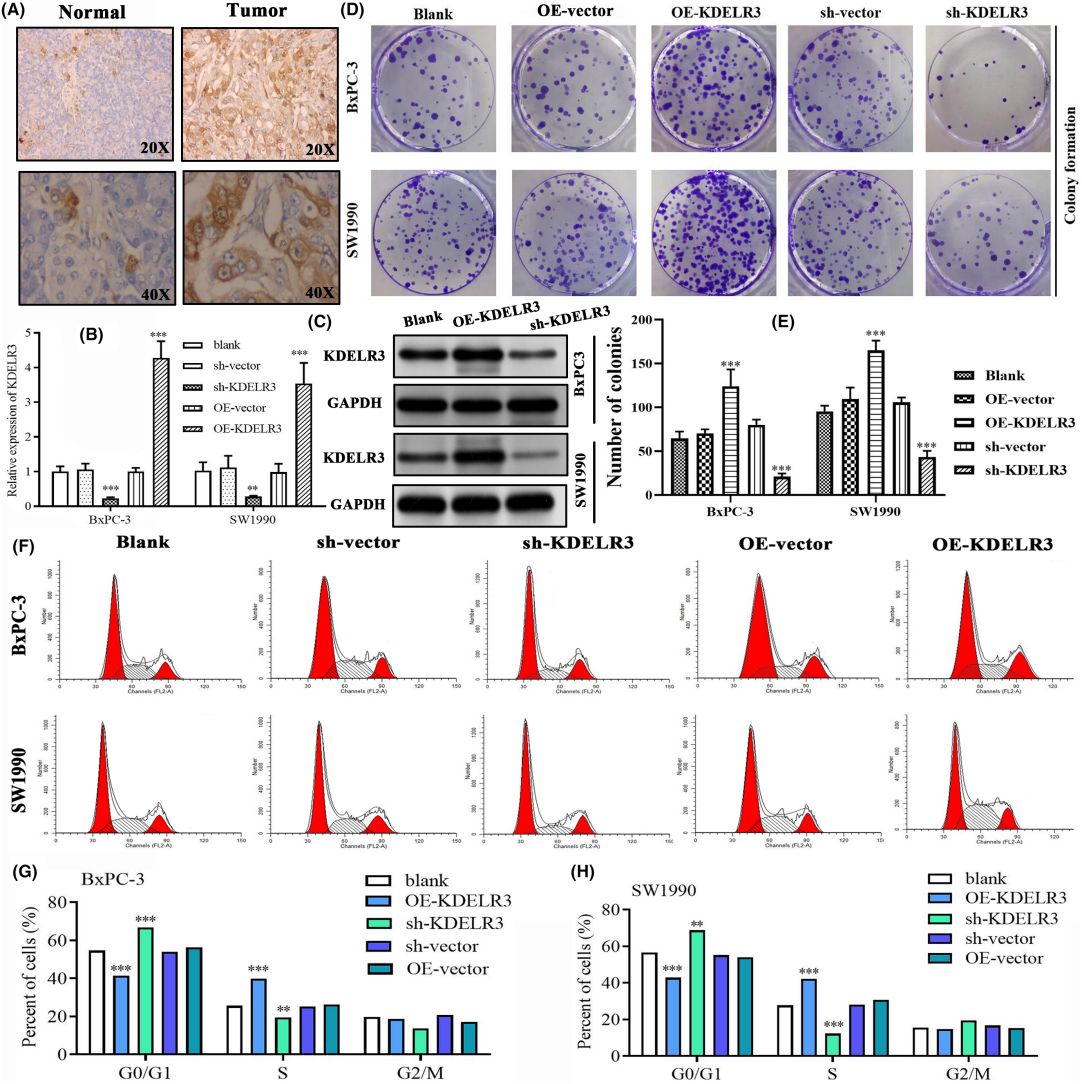
KDELR3 promotes the proliferation and regulates the cell cycle of PC cells. (A) Histological expression of KDELR3 in tumour and adjacent normal tissues through IHC detection (B, C) The transfection efficiency of sh‐KDELR3 and OE‐KDELR3 constructs. (D, E) The results of the colony formation assays revealed the effects of sh‐KDELR3 and OE‐KDELR3 on cell proliferation. (F–H) Flow cytometric analysis revealed that KDELR3 overexpression increased the proportion of cells at the S phase and decreased the proportion of cells at the G0/G1 phase. IHC, immunohistochemical staining; OE, overexpression; sh, short hairpin RNA. *p < 0.05, **p < 0.01, ***p < 0.001.

In this study, 20 pairs of PC and adjacent non‐cancerous tissues were collected to verify the expressive trends of KDELR3 and YWHAZ. qRT‐PCR analyses revealed that the *KDELR3* and *YWHAZ* in tumour samples were significantly higher than those in non‐cancerous samples (Figure S5B,C). Meanwhile, the expressions of KDELR3 and YWHAZ in PC cells (SW1990 and BxPC‐3 cells) were markedly upregulated when compared with that in healthy pancreatic cells (HPDE6‐C7 cells) (Figure S6A,B). IHC detections on 2 pairs of clinical samples also supported above findings (Figure 10A). sh‐KDELR3 and OE‐KDELR3 effectively altered the mRNA and protein levels of *KDELR3* in PC cell lines (Figure 10B,C). The number of colonies in the OE‐KDELR3‐transfected group was higher than that in the other groups, indicating that KDELR3 overexpression upregulated the proliferation of PC cells (Figure 10D,E). In contrast, cell proliferation was downregulated in the sh‐KDELR3‐transfected group (Figure 10D,E). Moreover, KDELR3 overexpression and knockdown exerted contrasting effects on the cell cycle of PC cells. Flow cytometric analysis revealed that KDELR3 overexpression significantly promoted the transition of cell cycle from the G0/G1 phase to the S phase, whereas *KDELR3* knockdown arrested the cell cycle at the G0/G1 phase (Figure 10F–H).

**FIGURE 11:**
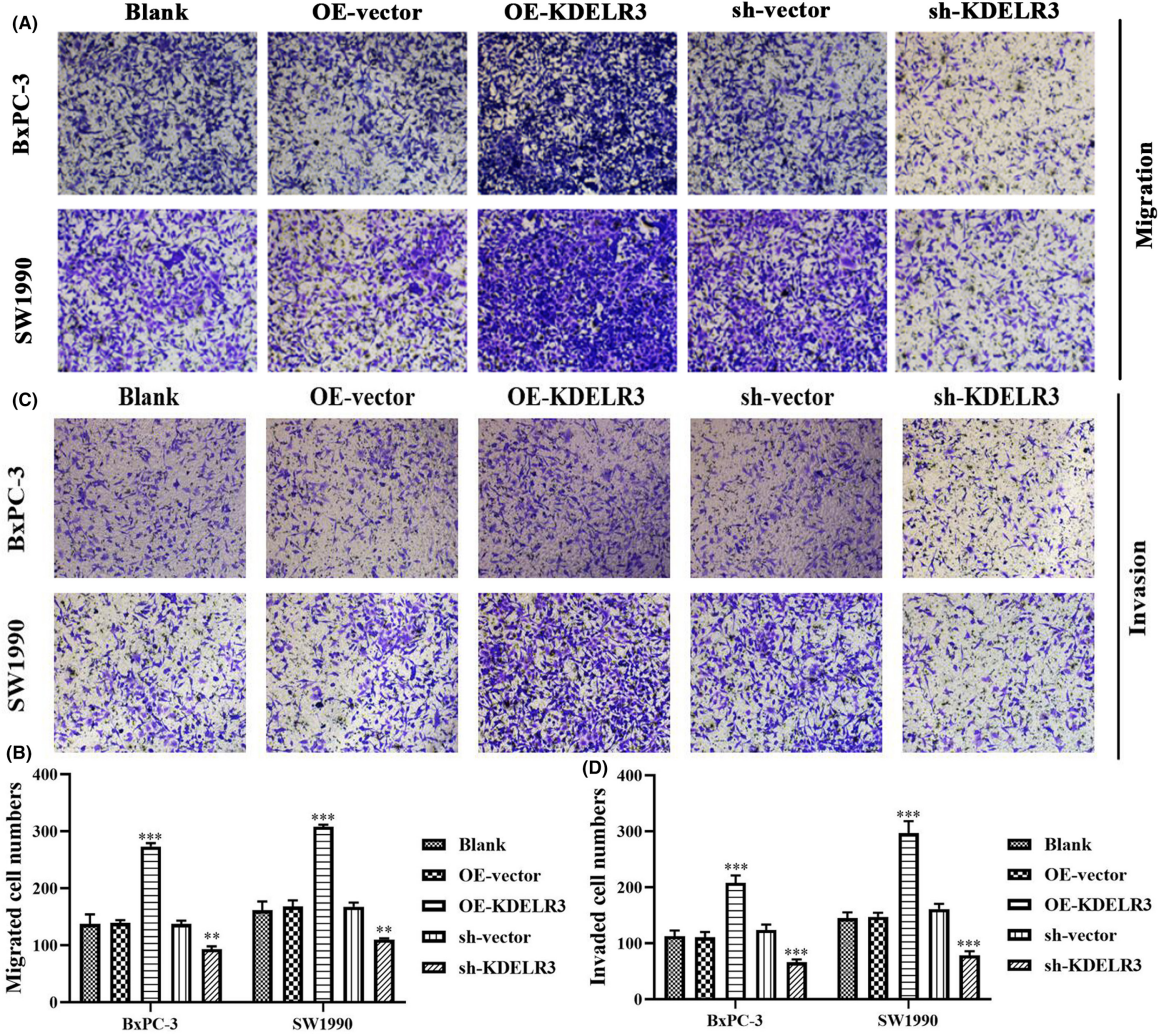
KDELR3 enhances the migration and invasion of PC cells. (A) Transwell migration assays were performed to evaluate cell migration in different groups. (B) The numbers of migrated cells in different groups. (C) Transwell invasion assays were performed to evaluate cell invasion in different groups. (D) The counts of invasive cells in different groups. OE, overexpression; PC, pancreatic cancer; sh, short hairpin RNA. *p < 0.05, **p < 0.01, ***p < 0.001.

KDELR3 upregulated the migration and invasion of PC cells. The numbers of migrated cells in the OE‐KDELR3‐transfected group were significantly higher than those in other groups. In contrast, the number of migrated cells was the least in the sh‐KDELR3‐transfected group (Figure 11A,B). The results of the Transwell invasion assays revealed that KDELR3 overexpression markedly increased the invasive ability of BxPC‐3 and SW1990 cells (Figure 11C,D). In contrast, *KDELR3* knockdown downregulated cell invasion (Figure 11C,D). These findings suggest that KDELR3 promotes PC progression.

### 3.12 *YWHAZ* also has promotive effects on the malignant behaviours of pancreatic carcinoma cells

**FIGURE 12:**
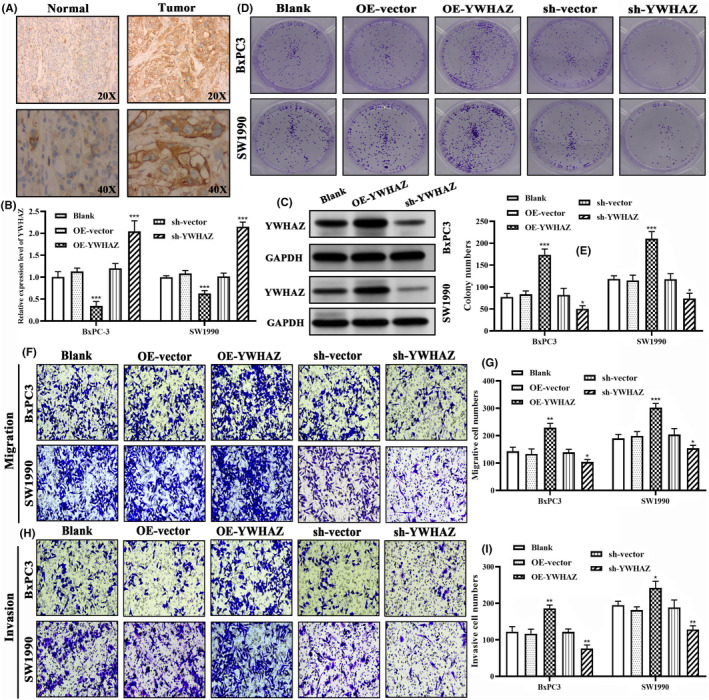
YWHAZ enhances the malignant potentials of PC cells. (A) Histological expression of YWHAZ in tumour and adjacent normal tissues through IHC detection (B, C) The transfection efficiency of sh‐YWHAZ and OE‐YWHAZ constructs. (D, E) The results of the colony formation assays revealed the effects of sh‐YWHAZ and OE‐ YWHAZ on cell proliferation. (F) Transwell migration assays were performed to evaluate cell migration in different groups. (G) The numbers of migrated cells in different groups. (H) Transwell invasion assays were performed to evaluate cell invasion in different groups. (I) The counts of invasive cells in different groups. IHC, immunohistochemical staining; OE, overexpression; sh, short hairpin RNA. *p < 0.05, **p < 0.01, ***p < 0.001.

As for YWHAZ, IHC detection confirmed that YWHAZ was upregulated in tumour samples compared to normal ones (Figure 12A). Specific overexpression vector and shRNA could effectively alter the mRNA expressions of YWHAZ in BxPC3 and SW1990 cells (Figure 12B). The effectiveness of above genetic tools was also validated through western blot assays (Figure 12C). Clonogenic assays revealed that overexpression of YWHAZ promoted the proliferation of PC cells, whereas silencing YWHAZ inhibited this process (Figure 12D,E). Moreover, PC cells with YWHAZ overexpression exhibited more aggressive migratory and invasive capacities through Transwell assays (Figure 12F–H). Quantitative data analyses also confirmed that migrative and invasive cells in overexpression groups were significantly higher than that in control groups, but stained cells in silencing groups were obviously decreased (Figure 12G–I). Clearly, YWHAZ enhanced the malignant potentials of PC cells.

### 3.13 *KDELR3* knockdown suppresses tumour growth in the xenograft model

**FIGURE 13:**
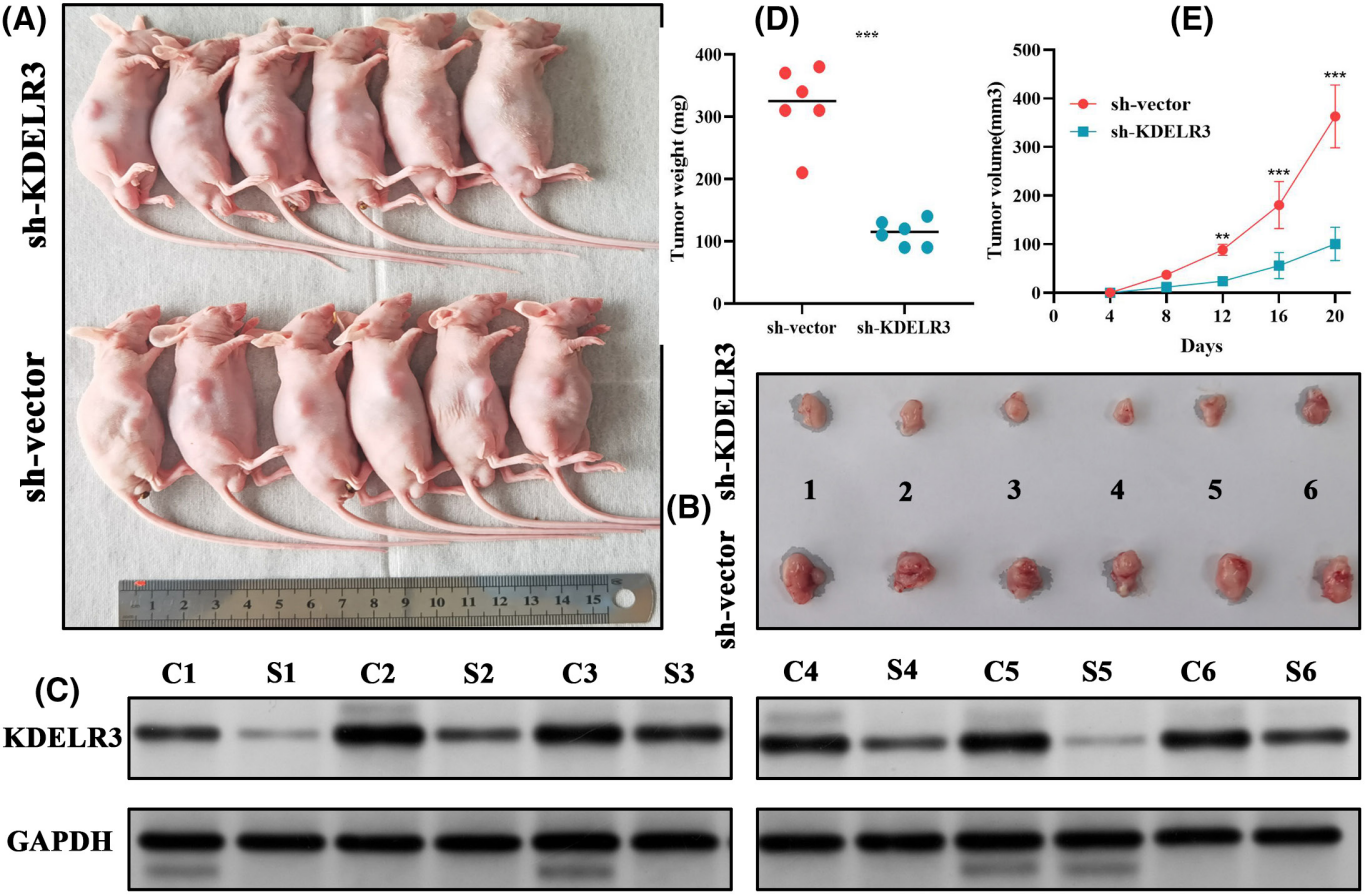
The effect of KDELR3 on xenograft tumour behaviour. (A, B) *KDELR3* knockdown suppresses PC growth in the xenograft model. (C) Expression levels of *KDELR3* in mice xenografts. C, negative control group, sh‐vector; S, *KDELR3* deletion group, sh‐KDELR3. (D) The differential tumour weight between mice injected with sh‐vector‐transfected cells and those injected with sh‐KDELR3‐transfected cells. (E) The differential tumour volume between mice injected with sh‐vector‐transfected cells and those injected with sh‐KDELR3‐transfected cells. PC, pancreatic cancer; sh, short hairpin. *p < 0.05, **p < 0.01, ***p < 0.001.

The tumour burden in nude mice injected with sh‐KDELR3‐transfected cells was lower than that in nude mice injected with negative control‐transfected cells (Figure 13A). Mice were sacrificed to obtain the xenograft tumour. *KDELR3* knockdown markedly suppressed xenograft tumour growth (Figure 13B). Western blot test manifested that the protein expressions of KDELR3 in xenograft tumours from the KDELR3 deletion group (sh‐KDELR3, S1‐S6) were significantly lower than that in the negative control group (sh‐vector, C1‐C6) (Figure 13C), which ensured the accuracy of the experiments. Tumour weight and volume in mice injected with sh‐KDELR3‐transfected cells were significantly lower than that in mice injected with sh‐vector‐transfected cells (Figure 13D,E). These findings indicate that *KDELR3* knockdown suppresses PAAD growth.

### 3.14 Potential upstream regulatory mechanisms of KDELR3

**FIGURE 14:**
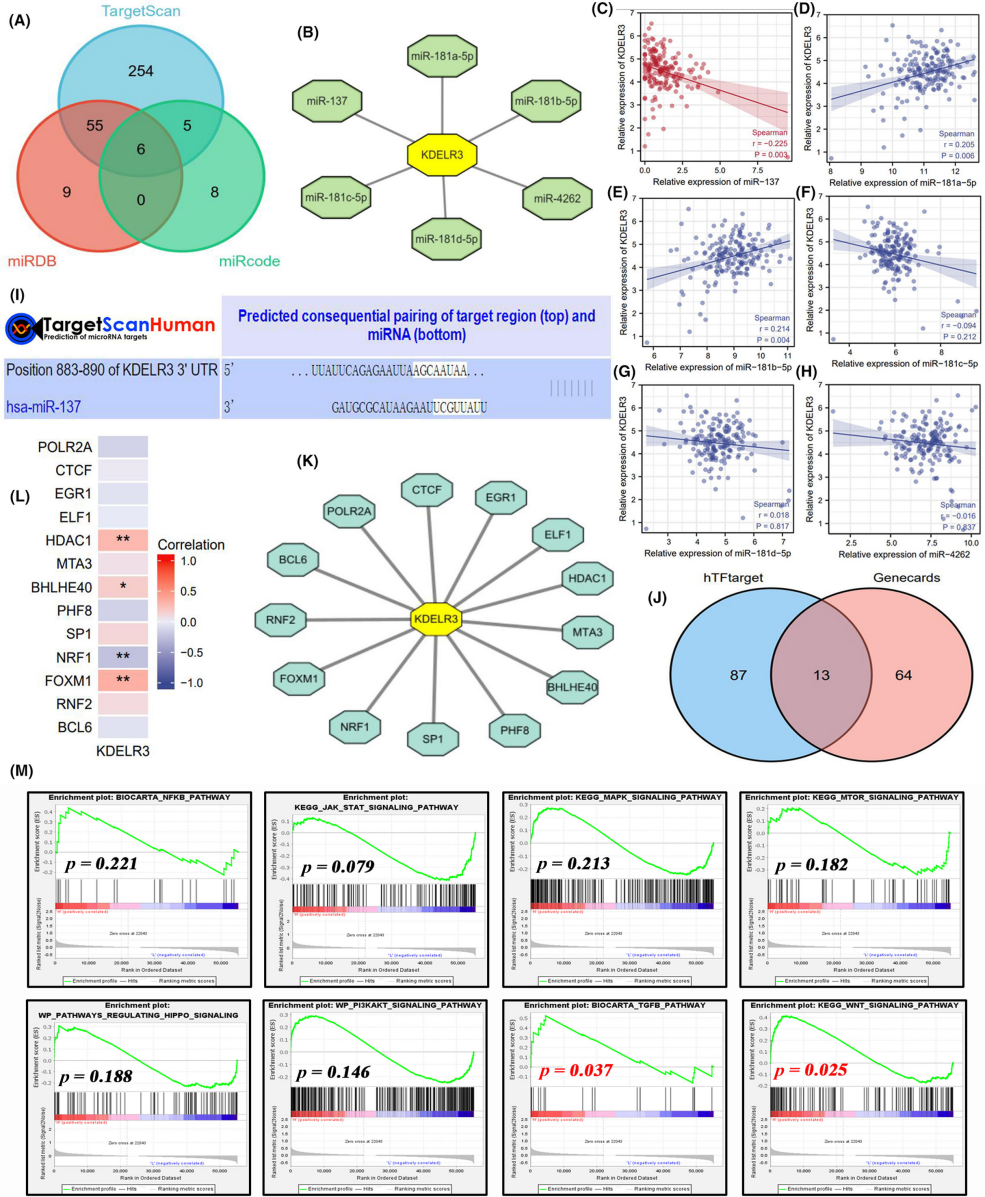
Potential upstream regulatory mechanisms of KDELR3. (A) Six candidate miRNAs were identified from three public databases. (B) Regulatory network of six predicted miRNAs. (C–H) Correlation of the expression of six predicted miRNAs with that of KDELR3. (I) Predicted binding site between miR‐137 and KDELR3. (J) Thirteen candidate TFs were identified from two public databases. (K) Regulatory network of 13 predicted TFs. (L) Correlation of the expression of 13 predicted TFs with that of KDELR3. (M) Potential effects of KDELR3 on the activities of eight classic tumour‐related signalling pathways based on GSEA analyses. miRNA, microRNA; TF, transcription factor. *p < 0.05; **p < 0.01.

This study examined the potential upstream regulatory mechanisms of KDELR3. The upstream miRNAs of KDELR3 were predicted. Six candidate miRNAs were identified from three miRNA databases (Figure 14A), The regulatory network of these miRNAs is shown in Figure 14B. miRNAs negatively regulate downstream target genes by binding to their 3′‐untranslated region (UTR). Thus, the candidate miRNAs were also hypothesized to negatively regulate *KDELR3*. Correlation analyses revealed that miR‐137 negatively regulated *KDELR3* (Figure 14C–H, Cor = −0.225, *p* = 0.003). Meanwhile, the binding site of miR‐137 in *KDELR3* was predicted using the TargetScanHuman database (Figure 14I). The findings of this study indicated that miR‐137 suppresses *KDELR3* expression by targeting its 3′‐UTR (5′‐AGCAAUAA‐3′).

The upstream TFs of KDELR3 were obtained from the hTFtarget and Genecards databases. In total, 13 TFs were predicted as the potential regulators of KDELR3. The regulatory network of these TFs was mapped (Figure 14J,K). As TFs activate the transcription of downstream genes, the correlation of their expression with KDELR3 expression was examined. The expression levels of HDAC1, BHLHE40 and FOXM1 were positively correlated with those of KDELR3. FOXM1 exhibited the highest correlation coefficient (Cor = 0.342, Figure 14L).

Moreover, GSEA analysis was utilized to explore the potential signalling pathways of KDELR3 in PAAD progression. The results revealed that Wnt and TGF‐β signalling pathways significantly enriched in patients with high KDELR3 expression, whereas the enrichments of other signalling pathways were not subjected to KDELR3 expression levels (Figure 14M).

### 3.15 miR‐137 mediates the PC progression through inhibiting *KDELR3*

**FIGURE 15:**
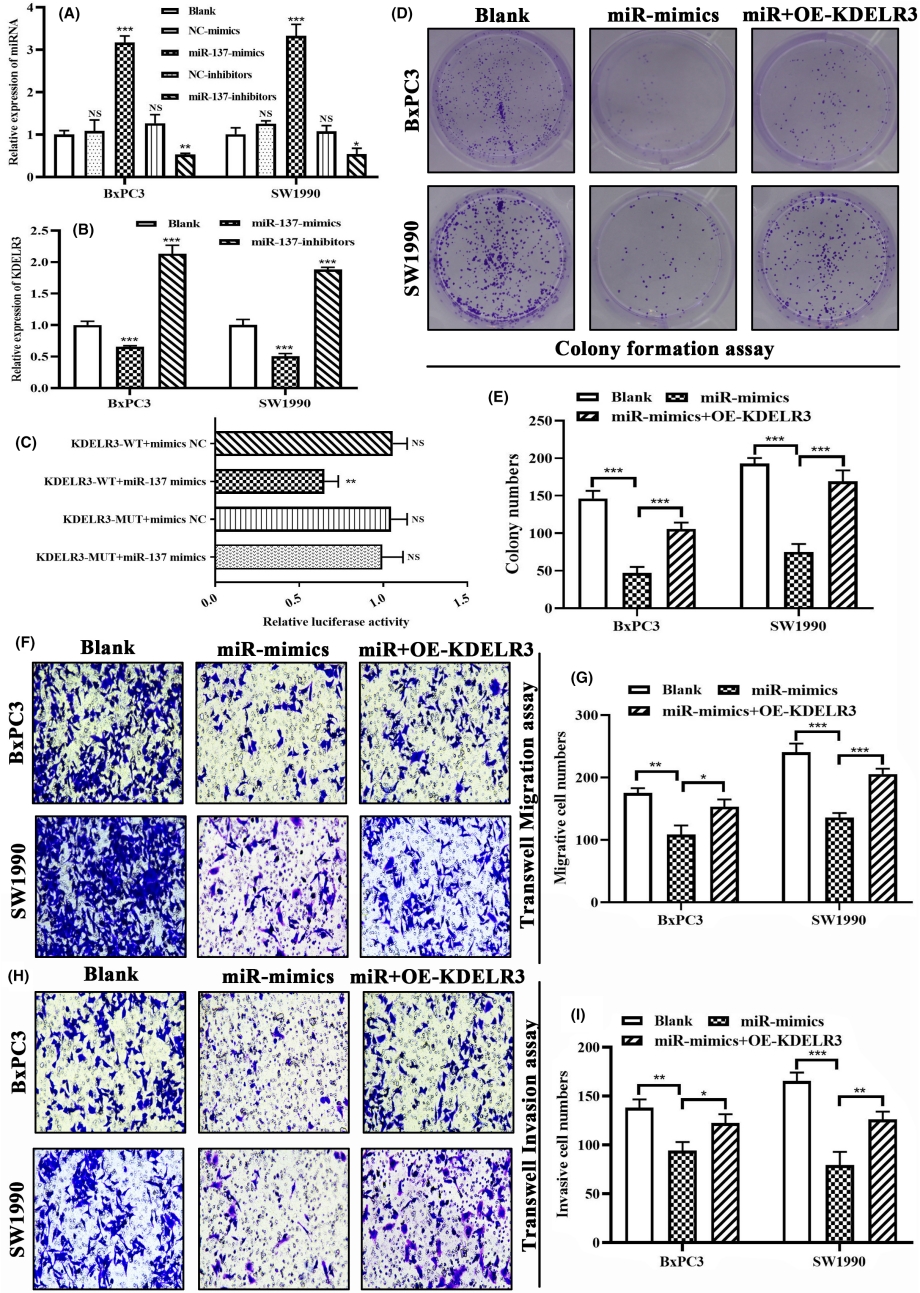
miR‐137 regulates PC progression through inhibiting KDELR3. (A) The transfection efficiency of miR‐137 mimics and inhibitors. (B) miR‐137 negatively regulates KDELR3 expression in PC cells. (C) The results of dual‐luciferase assays. (D, E) The effects of miR‐137‐KDELR3 axis on the proliferation of PC cells. (F, G) The effects of miR‐137‐KDELR3 axis on the migration of PC cells. (H, I) The effects of miR‐137‐KDELR3 axis on the invasion of PC cells. NS, no statistical significance; *p < 0.05, **p < 0.01, ***p < 0.001.

It was speculated that miR‐137 could pre‐transcriptional inhibit KDELR3 through a series of bioinformatic analyses. Subsequently, we conducted multiple experiments in vitro to demonstrate above hypothesis. PCR tests confirmed that the mimics and inhibitors of miR‐137 could effectively manipulate its expressions in BxPC3 and SW1990 cells (Figure 15A). Meanwhile, miR‐137 mimics significantly downregulated *KDELR3* expressions, whereas its inhibitors increased *KDELR3* expressions, indicating miR‐137 could negatively regulate *KDELR3* expression (Figure 15B). The dual‐luciferase analysis manifested that the luciferase intensity was significantly reduced when cells were co‐transfected with the WT recombinant vector of *KDELR3* and miR‐137 mimics, confirming that miR‐137 could bind to the 3′‐UTR region of *KDELR3* (5′‐AGCAAUAA‐3′, Figure 15C). Clonogenic assays revealed that miR‐137 mimics significantly weakened the proliferative abilities of PC cells, but overexpression of *KDELR3* could partially reverse above inhibitory effects (Figure 15D,E). Similarly, the cells transfected with miR‐137 mimics also exhibited attenuated migrative and invasive performance, while overexpression of *KDELR3* partially recovered the malignant capacities of PC cells (Figure 15F–I). All these findings depicted an upstream regulatory mode of *KDELR3*, namely miR‐137 retarded PC progression through targeting *KDELR3*.

## 4 DISCUSSION

PC is a major health and economic burden for society. The median survival time of patients with PC is <28 months owing to the high malignancy and metastasis of PC. Currently used treatments are not effective against PC. In response to ERS, the UPR process and various biological processes, such as cell proliferation, oncogene stimulation and mutation induction, are activated in the cells.^52^ ERS and UPR exhibit pleiotropic functions in cancer progression.^21^ The roles of ERS and UPR in the immune process, prognosis and progression of PAAD have not been elucidated. This study aimed to evaluate the roles of ERS and UPR in PAAD pathogenesis.

Prognostic assessment is crucial for managing patients with cancer. The currently used prognostic evaluation system is associated with some limitations. The American Joint Committee on Cancer eighth edition staging system cannot predict the prognosis of patients with T stage/lymph node‐negative PAAD.^53^ Although the eighth edition of the TNM staging system addresses the issues of extrapancreatic extension, its prognostic accuracy is not superior to that of the seventh edition of the TNM staging system.^54^ This study constructed a novel ERS risk signature, which increased the decision benefit of the traditional TNM staging system (Figure 4H). In addition to serving as an independent prognostic factor of PAAD, the ERS risk score can distinguish the differential prognoses of patients with no metastasized lymph nodes (N0 stage) (Figure 4P). These characteristics addressed the limitations of the TNM system. Therefore, the ERS risk score can accurately predict the prognosis of patients with PAAD.

Several studies have reported that cancer cells in which ERS is induced can modulate immune cell function although the underlying mechanisms have not been elucidated.^55^ ERS and UPR are associated with the TME. For example, ERS promotes the development of immune tolerance by activating immunosuppressive molecules and stimulating the accumulation and immunosuppressive functions of myeloid‐derived suppressor cells.^56^, ^57^ In this study, the enrichment of CD8 T cells and the cytotoxic effects of T cells were significantly downregulated in the high‐risk group (Figure 7A,B). The increased infiltration levels of macrophages in the high‐risk group promoted the onset of immunological tolerance (Figure 7A, Table 3). Thus, the ERS risk score can predict the immune status of patients with PAAD.

ERS can serve as a predictive biomarker for cancer immunotherapy response. Cell and animal experiments have demonstrated that the inhibition of UPR mediators exerts anti‐cancer effects.^56^ This study investigated the correlation between ERS risk score and the efficacy of ICB therapy from multiple perspectives. Although the analytical results were not consistent, the findings of this study suggested that a high ERS risk score adversely affects the efficacy of ICB therapy. This can be attributed to two main reasons. First, TMB may be the consequence of ICB therapy response and not the cause.^58^ Chen et al.^59^ reported that camrelizumab response was not correlated with TMB. Second, the efficacy of immunotherapy is dependent on the immune status of patients. The anti‐cancer immune processes were suppressed in the high‐risk group (Figure 7). Previous studies have reported that ERS adversely affects the efficacy of immunotherapy by modulating the TME.^6^ Therefore, a high ERS risk score is an unfavourable biomarker for ICB efficacy.

Metabolic reprogramming is a core hallmark of cancer. This study demonstrated that the metabolic pathways in PC, especially glycolysis, varied between the high‐risk and low‐risk groups. In tumours, the metabolism is reprogrammed toward glycolysis (‘Warburg effect’).^60^ The roles of glycolysis in prognosis and TIM in various tumours have been previously examined.^61^, ^62^, ^63^, ^64^ As glycolysis provides the biological precursors required for cell proliferation and confers therapy resistance to tumours,^63^ active glycolysis can drive cancer development. The glycolysis process was significantly enriched in the high‐risk group (Figure 6A), which may be the metabolic driver contributing to poor prognosis in high‐risk patients.

This study demonstrated that oncogenic mutations were upregulated in the high‐risk group. The mutation frequencies of *KRAS*, *TP53* and *CDKN2A* in the high‐risk group were higher than those in the low‐risk group (Figure 8F,G). *KRAS* mutation is a critical genetic alteration with diagnostic value in various tumours, such as PC, LUAD and colorectal cancer.^65^ Cheng et al.^66^ reported that *KRAS* mutation was closely associated with the upregulation of circulating regulatory T cells and indicated poor prognosis and advanced clinical stage in PAAD. Patients with *CDKN2A* mutation are at a high risk of developing melanomas and PC.^67^ The risk of cancer‐related death in patients with melanoma harbouring *CDKN2A* mutation is three times higher than that in patients with melanoma not harbouring *CDKN2A* mutation.^68^ The roles of *TP53* and its mutation in cancer have been previously reported.^69^, ^70^, ^71^ These findings indicate that the characteristic mutations in the high‐risk group are the critical epigenetic factors involved in PC progression.

KDELR3 encodes a member of the KDEL ER protein retention receptor family. The ER is a key site for the synthesis of biomacromolecules, such as proteins and lipids. Hence, KDELR3 is involved in the pathogenesis of various human diseases. For example, KDELR3 may serve as a diagnostic and therapeutic biomarker for AS.^14^ Limited studies have examined the functions of KDELR3 in cancer. Marie et al. demonstrated that KDELR3 deletion impairs the metastasis of malignant melanoma.^72^ This study, for the first time, demonstrated the cancer‐promoting properties of KDELR3 in PAAD, providing novel insights into the mechanisms underlying PAAD progression. Bai et al. reported that KDELR3 was significantly upregulated in HCC cells,^73^ which was consistent with its expression pattern in PC. This suggested that KDELR3 exerts oncogenic effects in various cancers.

This study has several limitations. The efficacy of the ERS risk signature should be evaluated in a real clinical cohort. Additionally, the effects of ERS risk signature on the metabolism and immune microenvironment of PAAD were examined using experimental approaches. Furthermore, the specific oncogenic mechanism of KDELR3 must be elucidated. There are currently several algorithms to construct prognostic model, such as cox regression analysis, ridge regression dimensionality reduction, elastic net regression analysis, random survival forest algorithm and lasso regression analysis. The optimal algorithm for constructing ERS risk signature cannot be determined yet. These limitations must be addressed in future studies.

## 5 CONCLUSIONS

As targeting ERS and UPR can potentially widen anti‐cancer strategies, this study constructed a novel ERS risk signature. Additionally, the functions of ERS risk signature in PAAD were evaluated from multiple perspectives, including the evaluation of prognostic value, immune process, metabolic reprogramming, therapeutic correlation and genomic information. Comprehensive analysis revealed that the ERS risk score can predict survival outcomes and immune landscapes. Furthermore, the ERS risk score predicted the efficacy of ICB therapy. Some characteristic oncogenic mutations, such as *KRAS* and *TP53* mutations, were observed in the high‐risk group. Limited studies have reported the role of KDELR3 and YWHAZ in PC. This study, for the first time, demonstrated their pro‐oncogenic activities in PC progression using in vitro and in vivo experiments. Mechanistically, a novel regulatory axis miR‐137/KDELR3 was also identified and validated. The findings of this study provided novel insights for the clinical assessment and mechanism elucidation of PAAD.

## AUTHOR CONTRIBUTIONS

**Shanshan Liu:** Data curation (lead); formal analysis (lead); investigation (lead); methodology (lead); resources (equal); software (lead); validation (lead); visualization (lead); writing – original draft (lead); writing – review and editing (equal). **Kaini He:** Data curation (lead); formal analysis (lead); investigation (lead); methodology (lead); resources (equal); software (supporting); validation (lead); visualization (lead); writing – original draft (lead); writing – review and editing (equal). **Longbao Yang:** Data curation (lead); formal analysis (lead); investigation (supporting); methodology (equal); resources (lead); software (lead); validation (equal); visualization (lead); writing – original draft (lead); writing – review and editing (supporting). **Fangshi Xu:** Data curation (supporting); formal analysis (supporting); investigation (equal); methodology (equal); resources (supporting); software (equal); validation (equal); visualization (lead); writing – original draft (supporting). **Xiaoguang Cui:** Data curation (supporting); formal analysis (equal); methodology (supporting); resources (supporting); software (equal); validation (equal); visualization (supporting); writing – original draft (supporting). **Li Qu:** Formal analysis (supporting); investigation (supporting); methodology (supporting); resources (supporting); software (supporting); validation (supporting); visualization (supporting). **Xueyi Li:** Conceptualization (equal); project administration (equal); resources (equal); supervision (equal); writing – review and editing (supporting). **Bin‐cheng Ren:** Conceptualization (lead); project administration (lead); resources (lead); supervision (lead); writing – original draft (equal); writing – review and editing (lead).

## FUNDING INFORMATION

No funding was received.

## CONFLICT OF INTEREST STATEMENT

The authors declare that they have no competing interests.

## Supporting information


Appendix S1

## Data Availability

The datasets used and/or analysed in the current study are available from the corresponding author upon reasonable request.
