# Supplementary material for: Endoplasmic reticulum stress regulators exhibit different prognostic, therapeutic and immune landscapes in pancreatic adenocarcinoma
Source: J Cell Mol Med. 2024 Feb 1;28(5):e18092. doi: 10.1111/jcmm.18092 (PMC10902308; doi:10.1111/jcmm.18092)
Supplement: Supplementary file 1 — Appendix S1 [file JCMM-28-e18092-s001.docx]

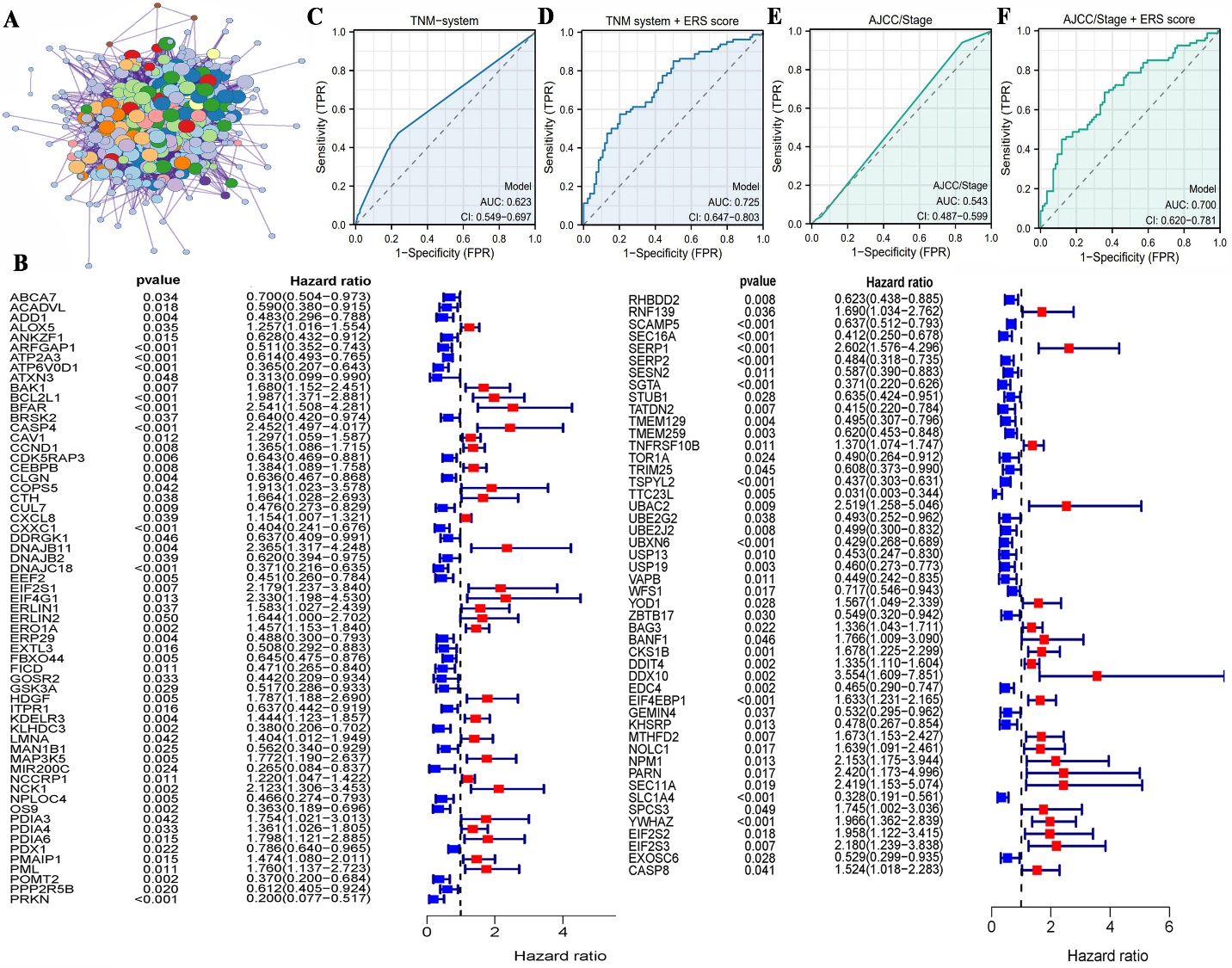


**Supplementary figure 1. ERS risk signature improves the predictive accuracy of AJCC-Stage and TNM models.** (A) PPI network of 379 ERS genes. (B) Identification of prognostic ERS genes through Cox regression analyses. (C-F) Combination of ERS risk score and traditional prognostic models increases predictive accuracy. PPI, protein-protein interaction.


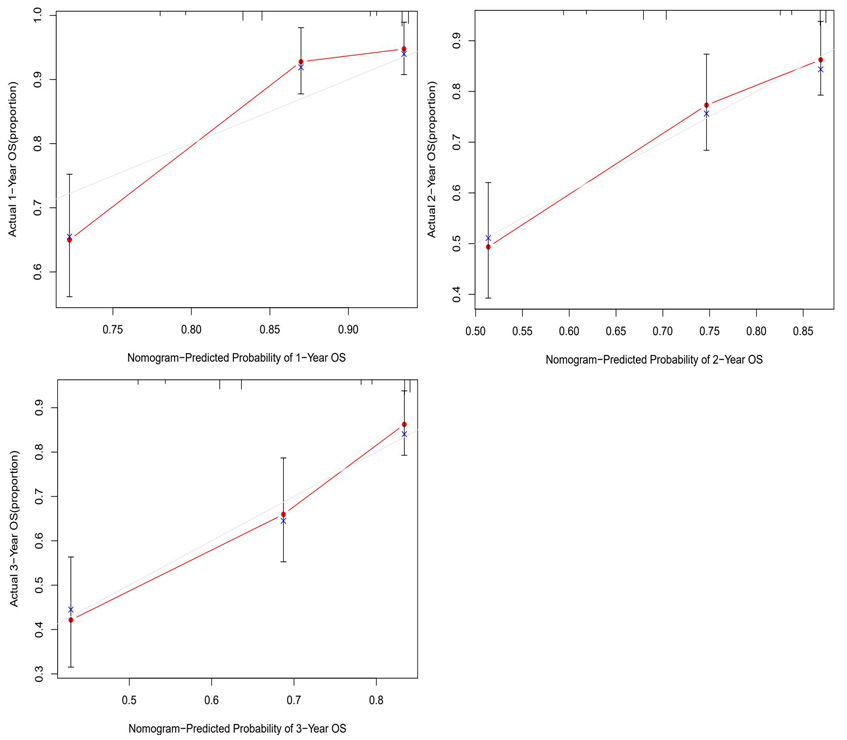


**Supplementary figure 2. The calibration plots for assessing the accuracy of ERS nomogram.**


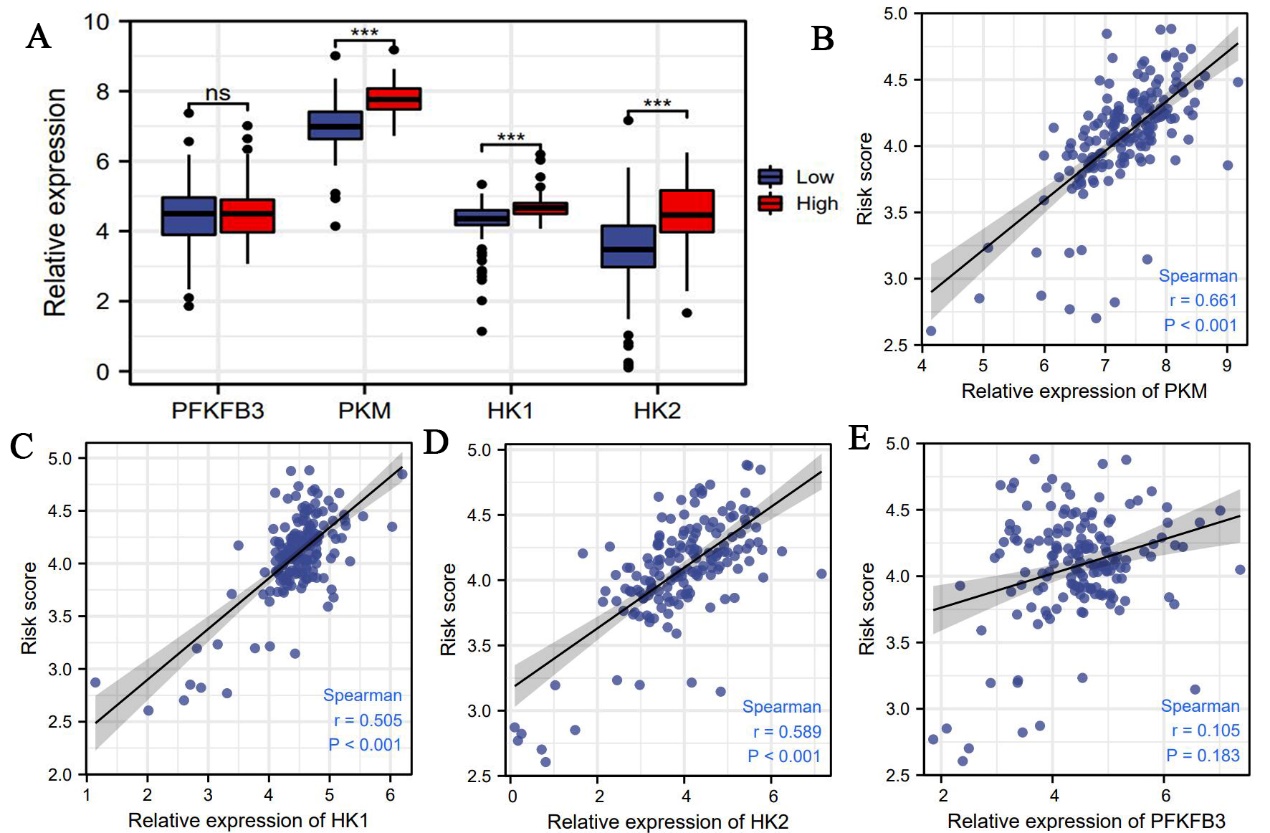


**Supplementary figure 3. Potential associations of ERS risk score with glycolysis rate-limiting enzymes.** (A) Differences in expressions of four glycolysis rate-limiting enzymes between high- and low-ERS risk groups. (B-E) Expressive correlations between ERS risk score and four glycolysis rate-limiting enzymes.


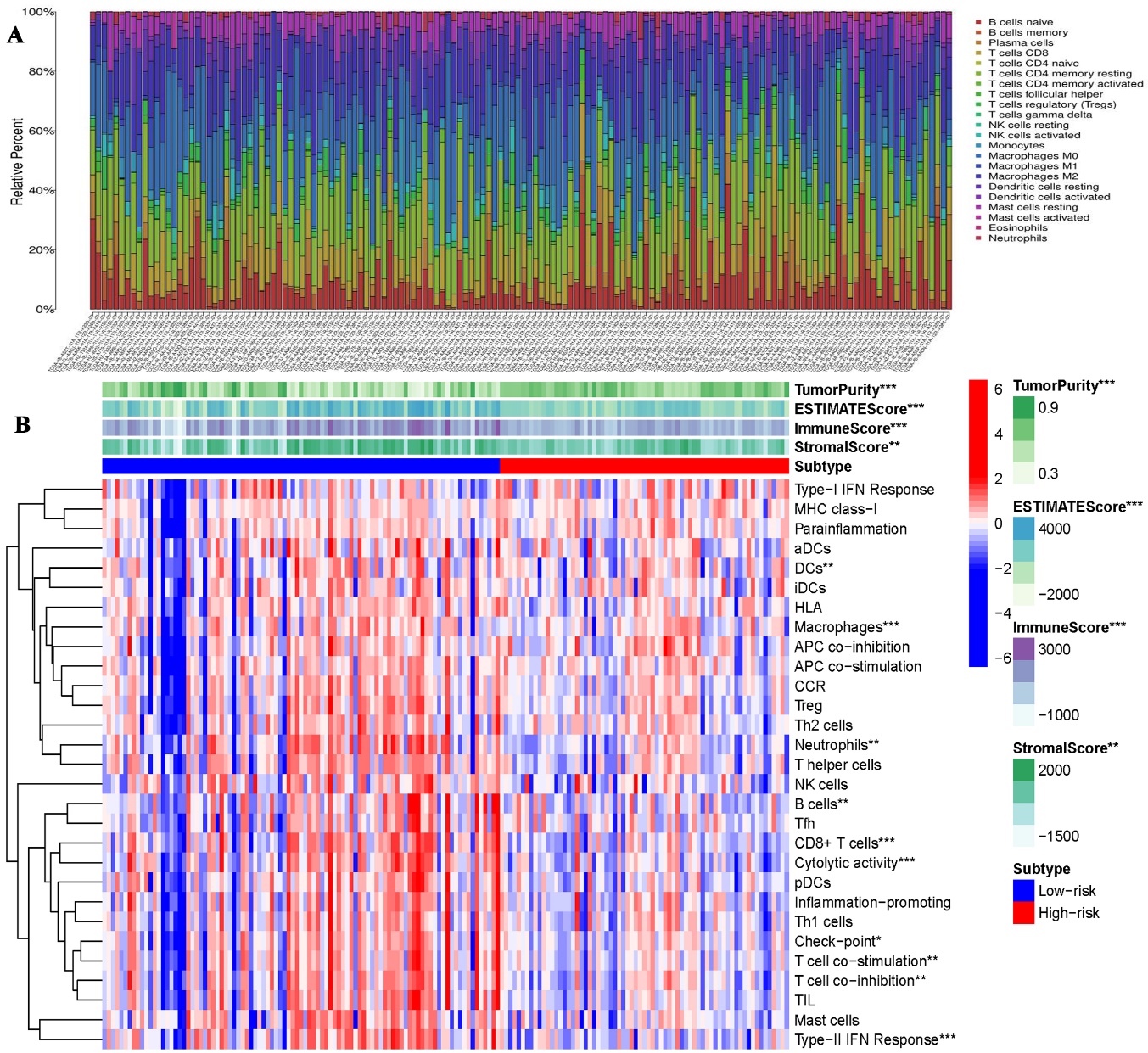


**Supplementary figure 4. The immune landscapes of different ERS-risk levels.** (A) The distribution of infiltration levels of 22 immune cells in each PAAD sample. (B) The immune heatmap of different ERS-risk levels.


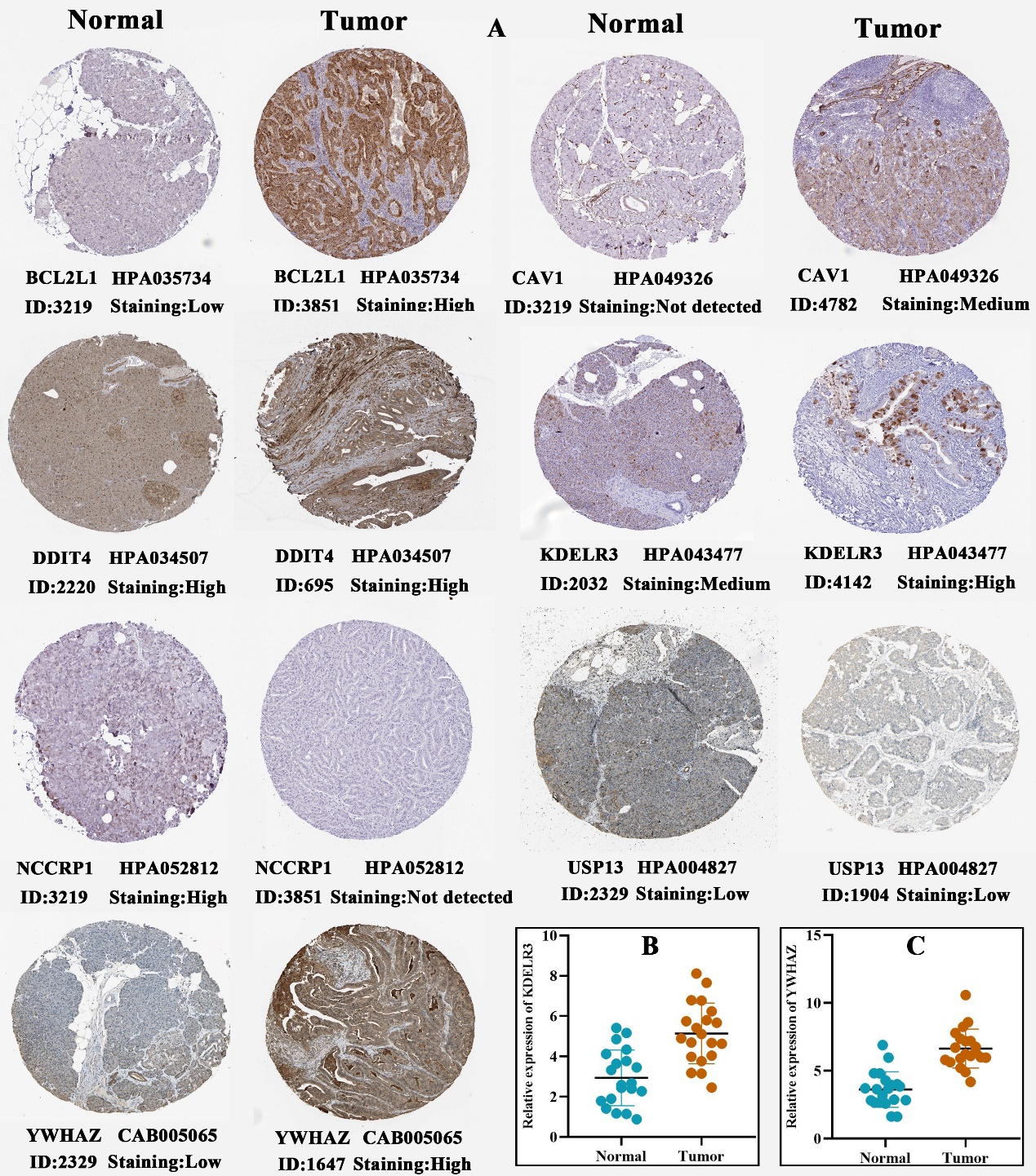


**Supplementary figure 5. The expressive status of seven ERS signature genes in PAAD.** (A) The histological expression levels of seven ERS signature genes curated in the HPA database. The name of the ERS-related gene, the antibody type, the patient ID, and the staining intensity are listed at the bottom of each image. (B-C) PCR detections on 20 pairs of clinical samples to confirm the expressive trends of KDELR3 and YWHAZ in PAAD. ERS, endoplasmic reticulum stress; HPA, Human Protein Atlas.


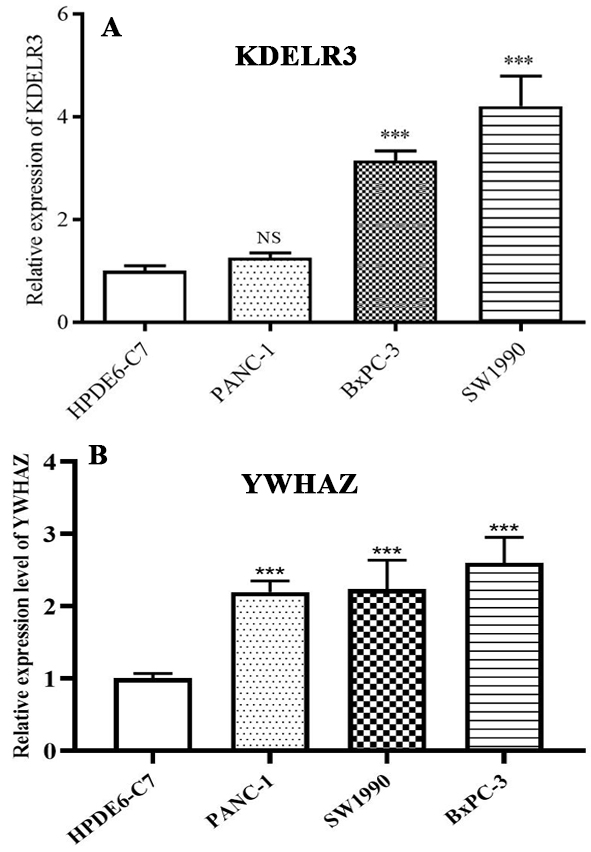


**Supplementary figure 6. The expressive difference of KDELR3 and YWHAZ between PC cells and normal pancreatic cells.**

Supplementary Table 1. The clinical characteristics of TCGA cohort

| Variables | Number (percentage) |
| --- | --- |
| Survival status |  |
| Alive | 86 (48.3%) |
| Dead | 92 (51.7%) |
| Age |  |
| ＜60 | 54 (30.3%) |
| ≥60 | 123 (69.1%) |
| Gender |  |
| Male | 98 (55.1%) |
| Female | 80 (44.9%) |
| Tumor Grade |  |
| G1 | 31 (17.4%) |
| G2 | 95 (53.4%) |
| G3 | 48 (26.8%) |
| G4 | 2 (1.2%) |
| Unknow | 2 (1.2%) |
| Clinical Stage |  |
| Stage I | 21 (11.8%) |
| Stage II | 146 (82.0%) |
| Stage III | 3 (1.7%) |
| Stage IV | 5 (2.8%) |
| Unknow | 3 (1.7%) |
| T stage |  |
| T1 | 7 (3.9%) |
| T2 | 24 (13.5%) |
| T3 | 142 (79.7%) |
| T4  Unknow | 3 (1.7%)  2 (1.2%) |
| M stage |  |
| M0 | 79 (44.4%) |
| M1 | 5 (2.8%) |
| Unknow | 94 (52.8%) |
| N stage |  |
| N0 | 50 (28.1%) |
| N1 | 123 (69.1%) |
| Unknow | 5 (2.8%) |

PAAD, Pancreatic adenocarcinoma; TCGA, The Cancer Genome Atlas.

Supplementary table 2. The clinical characteristics of ICGC cohorts.

| Variables | ICGC-PACA-AU | ICGC-PACA-CA |
| --- | --- | --- |
| Available samples | 81 | 194 |
| Survival status |  |  |
| Alive | 32 (39.5%) | 45 (23.2%) |
| Dead | 49 (60.5) | 149 (76.8%) |
| Age |  |  |
| ＜60 | 19 (23.5%) | 62 (31.9%) |
| ≥60 | 62 (76.5%) | 132 (68.1%) |
| Gender |  |  |
| Male | 40 (49.4%) | 84 (43.3%) |
| Female | 41 (50.6%) | 110 (56.7%) |
| Overall survival time (Year) | Mean=1.39 | Mean=1.97 |
| TNM-staging, Grade, and Clinical stage | Unknown | Unknown |

Supplementary table 3. The clinical characteristics of GEO cohorts

| Variables | GSE28735 | GSE57495 | GSE62452 |
| --- | --- | --- | --- |
| Available samples | 84 | 63 | 69 |
| Survival status |  |  |  |
| Alive | 26 (30.9%) | 21 (33.3%) | 16 (23.2%) |
| Dead | 58 (69.1%) | 42 (66.7%) | 49 (71.0%) / 4NA (5.8%) |
| Histological Grade | Unknown | Unknown |  |
| G1 | **/** | **/** | 2 (2.9%) |
| G2 | **/** | **/** | 35 (50.8%) |
| G3 | **/** | **/** | 30 (43.5%) |
| G4 | **/** | **/** | 1 (1.4%) |
| Unknown | **/** | **/** | 1 (1.4%) |
| Clinical stage | Unknown |  |  |
| Stage I | **/** | 13 (20.6%) | 4 (5.8%) |
| Stage II | **/** | 50 (79.4%) | 46 (66.7%) |
| Stage III | **/** | 0 (0%) | 13 (18.8%) |
| Stage IV | **/** | 0 (0%) | 6 (8.7) |
| Overall survival time (Year) | Mean=1.45 | Mean=1.99 | Mean=1.66 |
| Age, Gender, and TNM stages | Unknown | Unknown | Unknown |

NA, not available.

Supplementary table 4. 11 crucial ERS-related gene set from MSigDB database

| Names | Gene counts | Description |
| --- | --- | --- |
| GOBP Response to Endoplasmic Reticulum Stress | 256 | Any process that results in a change in state or activity of a cell (in terms of movement, secretion, enzyme production, gene expression, etc.) as a result of a stress acting at the endoplasmic reticulum. |
| GOBP Regulation of Response to Endoplasmic Reticulum Stress | 82 | Any process that modulates the frequency, rate or extent of response to endoplasmic reticulum stress. |
| GOBP Endoplasmic Reticulum Unfolded Protein Response | 75 | The series of molecular signals generated as a consequence of the presence of unfolded proteins in the endoplasmic reticulum (ER) or other ER-related stress. |
| GOBP Regulation of Endoplasmic Reticulum Unfolded Protein Response | 30 | Any process that modulates the frequency, rate or extent of endoplasmic reticulum unfolded protein response |
| Hallmark Unfolded Protein Response | 113 | Genes up-regulated during unfolded protein response, a cellular stress response related to the endoplasmic reticulum. |
| Reactome Unfolded Protein Response | 92 | Unfolded protein response. |
| WP Unfolded Protein Response | 25 | Unfolded protein response. |
| GOBP IRE1 Mediated Unfolded Protein Response | 19 | A series of molecular signals mediated by the endoplasmic reticulum stress sensor IRE1. |
| GOBP PERK Mediated Unfolded Protein Response | 18 | A series of molecular signals mediated by the endoplasmic reticulum membrane stress sensor PERK. |
| REACTOME PERK Regulates Gene Expression | 32 | PERK regulates gene expression. |
| GOBP ATF6 Mediated Unfolded Protein Response | 7 | A series of molecular signals mediated by the endoplasmic reticulum membrane stress sensor ATF6. |

Supplementary table 5. The ERS-related gene set in our study


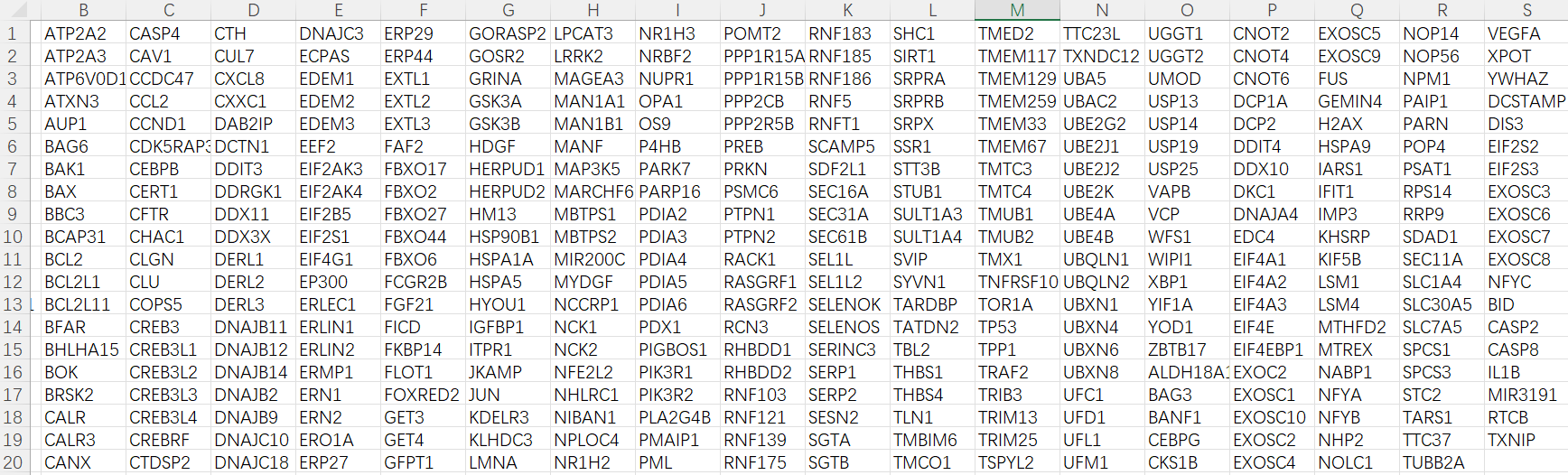


Supplementary table 6. The detailed description of the gene sets used in metabolic analyses.

| Names | Gene counts | Description |
| --- | --- | --- |
| GO glycolytic process | 106 | Fermentation that includes the anaerobic conversion of glucose to pyruvate via the glycolytic pathway. |
| Hallmark Glycolysis | 200 | Genes encoding proteins involved in glycolysis and gluconeogenesis. |
| KEGG Glycolysis Gluconeogenesis | 62 | Glycolysis / Gluconeogenesis |
| Reactome Glycolysis | 72 | Glycolysis |
| MODULE 337 | 64 | Nucleotide metabolism |
| WP Nucleotide Metabolism | 19 | Nucleotide metabolism |
| KEGG DNA Replication | 36 | DNA replication |
| KEGG Pentose Phosphate Pathway | 27 | Pentose phosphate pathway |
| KEGG Amino sugar and Nucleotide sugar Metabolism | 44 | Amino sugar and nucleotide sugar metabolism |
| Amino acid and derivative Metabolic Process | 101 | The chemical reactions and pathways involving amino acids, organic acids containing one or more amino substituents, and compounds derived from amino acids. |
| Reactome Glutamate and Glutamine Metabolism | 14 | Glutamate and glutamine metabolism |
| KEGG Glycine Serine and Threonine Metabolism | 31 | Glycine, serine and threonine metabolism |
| GOBP Lipid Homeostasis | 173 | Any process involved in the maintenance of an internal steady state of lipid within an organism or cell. |
| WP Lipid Metabolism Pathway | 29 | Lipid metabolism pathway |
| Hallmark Fatty acid Metabolism | 158 | Genes encoding proteins involved in metabolism of fatty acids. |
| Biosynthetic process | 470 | The energy-requiring part of metabolism in which simpler substances are transformed into more complex ones, as in growth and other biosynthetic processes. |

Supplementary Table 7. The specific sequences of sh-KDELR3 and OE-KDELR3

| Gene | Sequence (5' -> 3') |
| --- | --- |
| sh-KDELR3 | CCGGAGTAGTACAAACCATCTTCTACTCGAGTAGAAGATGGTTTGTACTACTTTTTTG |
| OE-KDELR3 | KDELR3-XbaI-F: **GCTCTAGA**ATGAACGTGTTCCGAATCCTCGGCG |
|  | KDELR3-EcoRI-R: **GGAATTC**TCAGATTGGCATTGGAAGACTTAAC |

OE, over expression.

Supplementary Table 8. The primer lists.

| Gene | Primer | Sequence (5' -> 3') |
| --- | --- | --- |
| KDELR3 | Forward | 5′- GAGGCTGAGACCATAACTACTC-3′ |
|  | Reverse | 5′- AGAAATTCTCAGTCTGGTACCG-3 |
| GAPDH | Forward | 5'‐GTCGCCAGCCGAGCCACATC‐3 |
|  | Reverse | 5'‐CCAGGCGCCCAATACGACCA‐3' |
